# Characterization of the Breast Cancer Liver Metastasis Microenvironment via Machine Learning Analysis of the Primary Tumor Microenvironment

**DOI:** 10.1158/2767-9764.CRC-24-0263

**Published:** 2024-10-31

**Authors:** Dylan A. Goodin, Eric Chau, Junjun Zheng, Cailin O’Connell, Anjana Tiwari, Yitian Xu, Polly Niravath, Shu-Hsia Chen, Biana Godin, Hermann B. Frieboes

**Affiliations:** 1 Department of Bioengineering, University of Louisville, Louisville, Kentucky.; 2 Department of Nanomedicine, Houston Methodist Research Institute, Houston, Texas.; 3 Immunomonitoring Core, Center for Immunotherapy Research, Houston Methodist Research Institute, Houston, Texas.; 4 Breast Medical Oncology Faculty, Houston Methodist Cancer Center, Houston, Texas.; 5 Department of Obstetrics and Gynecology, Weill Cornell Medical College, New York, New York.; 6 Department of Biomedical Engineering, Texas A&M University, College Station, Texas.; 7 UofL Health – Brown Cancer Center, University of Louisville, Louisville, Kentucky.; 8 Center for Predictive Medicine, University of Louisville, Louisville, Kentucky.

## Abstract

Breast cancer liver metastases (BCLM) are hypovascular lesions that resist intravenously administered therapies and have grim prognosis. Immunotherapeutic strategies targeting BCLM critically depend on the tumor microenvironment (TME), including tumor-associated macrophages. However, *a priori* characterization of the BCLM TME to optimize therapy is challenging because BCLM tissue is rarely collected. In contrast to primary breast tumors for which tissue is usually obtained and histologic analysis performed, biopsies or resections of BCLM are generally discouraged due to potential complications. This study tested the novel hypothesis that BCLM TME characteristics could be inferred from the primary tumor tissue. Matched primary and metastatic human breast cancer samples were analyzed by imaging mass cytometry, identifying 20 shared marker clusters denoting macrophages (CD68, CD163, and CD206), monocytes (CD14), immune response (CD56, CD4, and CD8a), programmed cell death protein 1, PD-L1, tumor tissue (Ki-67 and phosphorylated ERK), cell adhesion (E-cadherin), hypoxia (hypoxia-inducible factor-1α), vascularity (CD31), and extracellular matrix (alpha smooth muscle actin, collagen, and matrix metalloproteinase 9). A machine learning workflow was implemented and trained on primary tumor clusters to classify each metastatic cluster density as being either above or below median values. The proposed approach achieved robust classification of BCLM marker data from matched primary tumor samples (AUROC ≥ 0.75, 95% confidence interval ≥ 0.7, on the validation subsets). Top clusters for prediction included CD68^+^, E-cad^+^, CD8a^+^PD1^+^, CD206^+^, and CD163^+^MMP9^+^. We conclude that the proposed workflow using primary breast tumor marker data offers the potential to predict BCLM TME characteristics, with the longer term goal to inform personalized immunotherapeutic strategies targeting BCLM.

**Significance:**

BCLM tissue characterization to optimize immunotherapy is difficult because biopsies or resections are rarely performed. This study shows that a machine learning approach offers the potential to infer BCLM characteristics from the primary tumor tissue.

## Introduction

Breast cancer liver metastases (BCLM) are prognostically grim, with median overall survival of 20 months (1), presenting a critical need for more effective therapy. BCLM typically seem as small, hypovascular nodules under contrast imaging (2), which do not rely on angiogenesis by receiving nutrients mainly from surrounding hepatic capillaries. Accordingly, transport of intravenously injected therapeutics can be impaired due to physiologic drug resistance (3). To facilitate drug transport into tumors, targeting of macrophages has emerged as an immunotherapeutic strategy. Macrophages are a key phagocytic population of liver resident immune cells, taking up nanomaterials and frequently congregating around inflamed tumor lesions (4). Macrophages originate from monocyte (CD14^+^) lineage (5) or yolk sac–derived erythro-myeloid progenitors (6) and have been classified into a phenotypic spectrum ranging from antitumor (M1; CD68^+^ and CD163^−^CD206^−^) to protumor variants (M2; CD68^+^, CD163^+^, or CD206^+^; ref. 7).

We have previously shown that physiologic resistance to drug therapy in BCLM could in principle be overcome by targeting macrophages with nanotherapy (8). With nanoalbumin paclitaxel encapsulated into a solid multistage nanovector (nab-PTX-MSV), macrophages were shown to uptake significant numbers of nanovectors, acting as “depots” to locally release drug in the vicinity of BCLM. Efficacy of this approach, evaluated *in vitro* (9–11), *in silico* (9–12), and *in vivo* (8), depends on the BCLM tumor microenvironment (TME) characteristics and, particularly, the number and phenotype of associated macrophages. Contrary to the general notion that M2 macrophages solely enhance tumor resistance to therapy by favoring tumor growth, our *in silico* modeling validated through our prior studies underscored the potential role of M2 macrophages in sensitizing the TME to nanotherapy, suggesting that a balance between M1 and M2 phenotypes may be conducive to an optimal antitumor response (10, 11).

Defining immunotherapy parameters to optimally target BCLM would benefit from understanding the patient-specific BCLM TME characteristics. Growth of BCLM can be classified from [extracellular matrix (ECM)-preserving] replacement growth to less common (ECM-driven) desmoplastic growth (13). Spatially heterogeneous cancer cell proliferation [inferred from tumor cell expression of Ki-67 (14) and phosphorylated ERK (15)] as well as lesion vascularity (CD31; ref. 16), fosters viable and hypoxic tissue regions. Macrophage migration is facilitated toward hypoxic regions [e.g., via hypoxia-inducible factor-1α (HIF1α) as a chemoattractant; ref. 17] but can be inhibited by dense ECM (primarily comprised of collagen; ref. 18), promoted by fibrogenic activity [identified by alpha smooth muscle actin (αSMA); ref. 19] and breached by tumor cells via matrix metalloproteinase 9 (MMP9; ref. 20). Furthermore, PD-L1, a common inhibitor of CD8a T cell–mediated immunotherapy via the programmed cell death protein 1 (PD1)/PD-L1 axis, can be expressed in primary breast tumors by local macrophages (21). Macrophage activity can be influenced by helper (CD4^+^) T cells, which can exert protumor or antitumor effects (22). NK (CD56^+^) cells, an innate antitumor immune species common to the liver, also contribute to the tumor response (23).

Tissue availability to obtain information about BCLM characteristics for more efficient immunotherapeutic targeting is scarce because biopsy does not offer a survival benefit for late metastases (24), and resection is generally discouraged (25). In contrast, biopsy or resection from primary breast tumors is usually obtained, and histologic analysis is routinely performed (26). Ideally, BCLM TME characteristics could be inferred from the primary tumor tissue. Recent work provides evidence for this possibility, showing the feasibility of correlating the immune TME between matched primary colorectal cancer and liver metastases (27). This study hypothesized that the BCLM TME characteristics could be inferred from the matched primary tumor. To evaluate this hypothesis, imaging mass cytometry (IMC) analysis of 23 different markers (related to immune cells, tumor markers, hypoxia, vascularity, and ECM, as noted above) was performed on matched samples from patients with primary breast cancer and BCLM to associate the TME of these two locations using statistical and machine learning (ML) approaches.

## Materials and Methods

### Patient samples

Despite the scarcity of matched primary breast cancer and BCLM clinical samples, this study was able to acquire a set of patient-matched biopsies. De-identified patient-matched primary breast cancer and BCLM paraffin slide samples (*n* = 15 pairs) were commercially obtained from BioCoreUSA. Patient consent was not required. All patients were female, ranging in age from 28 to 69 and from 31 to 73 at collection of primary tumor and BCLM samples, respectively. Primary breast cancer samples were collected following mastectomy between 2015 and 2018, whereas matched BCLM were collected by needle core biopsy between 2018 and 2020. The number in each group was considered adequate for proof-of-concept testing (28) of the hypothesis that BCLM TME characteristics could be predicted via ML analysis of the primary TME. The sampled tissues had no specific clinicopathologic inclusion criteria aside from being patient-matched breast cancer and liver metastasis tissue samples.

### Sample staining analysis

All patient slides were deparaffined and stained with hematoxylin and eosin (Sigma). Slides were holistically imaged, under brightfield microscopy at 10× magnification (Nikon Eclipse 80i) for examination of specific regions of interest (ROI) to identify areas for IMC analysis. Due to the size of the histologic cores, five and three ROIs per slide were analyzed in matched primary breast and BCLM samples, respectively. ROIs were randomly selected based on the hematoxylin and eosin staining, focusing on areas of high nuclear density. Each ROI was chosen at random within unique tumors and dependent on slide tissue size. IMC analysis was performed on 0.7 mm × 0.7 mm tissue samples in each ROI.

### IMC

IMC allows simultaneous marker detection, making it suitable for profiling the TME. IMC data were preprocessed and checked for tissue integrity, staining quality, and signal range prior to analysis. For each ROI, single cells were segmented using *ilastik* (29) and *CellProfiler* (30), based on DNA staining (Ir191) and other cell surface markers. Following segmentation, data were processed using Histology Topography Cytometry Analysis Toolbox (31), in which mean marker intensities for single cells were extracted. Data were consolidated in R scripts for downstream analysis. Intensity values were clipped at 99.5 percentile to eliminate outliers and subsequently normalized to 0 to 1 range, giving equal weights to each marker. For samples from different tissue types, expression values were aligned using geometrical means of marker expression within the same tissue type before abovementioned data normalization to remove tissue type–specific background noise and to decrease batch effect. Normalized intensities were used to perform unsupervised clustering in Seurat (32) using *Louvain* algorithm (33). Cell clusters were annotated based on the mean expressions of markers and consolidated into 23 marker clusters denoting known cell phenotypes. Cell densities of each phenotype were calculated by normalizing counts by ROI. Neighborhood analysis identified statistically significant neighboring status for each pair of phenotypes (31). Neighborhood heatmaps normalized results to −1 to 1, in which 1 (or red) denotes that two phenotypes are neighboring each other, −1 (or blue) denotes significant separation, and 0 indicates no significant spatial relationship. IMC ROIs were excluded for subsequent analysis if signal lacked tumor-specific markers [Ki-67, αSMA, or E-cadherin (E-cad)].

### IMC data preprocessing

To prevent biasing due to tissue type, preprocessing steps were performed on breast and liver IMC data separately. Marker cluster densities were first transformed using base 10 logarithm to reduce heteroskedasticity (34). Each ROI was then scaled by total cluster intensities to control for differences in tissue mass represented per ROI. The IMC data were processed and clustered in two analytical batches. Thus, each batch (primary tumor and BCLM) was separately centered to focus on differences in expression (34). Finally, each cluster was averaged across ROIs on a per-patient basis to create one representative primary tumor and BCLM sample pair per patient.

### ML analysis

To predict relative cluster density expression in BCLM TME from the primary TME, a comprehensive ML analysis was performed across all BCLM cluster densities using *caret* package in R (v. 4.2.2). For each cluster, patients were separated into low (< median) or high (≥ median) expression groups. Multiple ML models were tested, including neural networks [neural networks with principal component step (*pcaNNet*), neural network (*nnet*), model averaged neural network (*avNNet*), multilayer perceptron with multiple layers (*mlpWeightDecayML*)], k-nearest neighbors (*knn*), naïve Bayes (*naive_bayes*), linear models [generalized (*glm*), boosted (*glmboost*)], random forests [random forest (*rf*), oblique random forest with SVM as splitting model (*ORFsvm*)], and support vector machines [linear kernel (*svmLinear*), radial basis function kernel (*svmRadial*), class weights (*svmRadialWeights*), and polynomial kernel (*svmPoly*)]. For each model, features were ranked using *varImp*, with all feature subsets tested from the top two primary tumor clusters as ranked by *varImp* to all primary tumor clusters. The calculation of variable importance by *varImp* included model-specific learning methods (e.g., random forest) and generalized ROC curve analysis, such as for naïve Bayes. Then, each model was re-trained on a feature subset generated by sequentially adding features in the order determined by *varImp* feature rankings. Five-fold cross-validation with 20 resampling iterations was performed to obtain a total of 100 unique permutations. Kappa was selected as the optimization metric for *caret* model training.

To evaluate classifications, AUROC was calculated for each BCLM cluster and feature number combination, along with variable importance data. Performance metrics were calculated as the average across all folds and resampling iterations. AUROC 95% confidence intervals (CI) were generated using *t* test distribution SE. A single AUROC-optimized model was selected for each cluster. F1 as the average of precision and recall was also computed. All plots were generated using *ggplot2* package (RRID: SCR_014601). Comparisons between differing ML feature rankings were calculated in terms of relative values because the scale of the rankings differs based on the ML model selected. To prevent ranking biases, clusters with equal variable importance were equally ranked.

### Statistical analysis

All statistical analyses were performed in R (v. 4.2.2). A comparison between marker cluster densities was performed with *t* test or Wilcoxon test depending on normality of the data per Shapiro–Wilk test with *P* ≤ 0.05 as threshold. Correlations were done with *corrplot* package using Pearson or Spearman correlations, depending on normality of the data per Shapiro–Wilk test. Strong correlation was defined as |*r*| ≥ 0.75 or |*ρ*| ≥ 0.75 for Pearson and Spearman, respectively. Bonferroni correction was applied to adjust for repeated t-test (for parametric data) or Wilcoxon rank sum test (for non-parametric data) across patient groups, with two-sided *P* value < 0.0025 (*P*-adj) considered significant. Partial least squares discriminant analysis (PLS-DA) was performed using *plsda* function from *mdatools* package.

### Data availability

The data generated in this study are available upon request from the corresponding authors.

## Results

### Patient characteristics and study methodology

**Figure 1:**
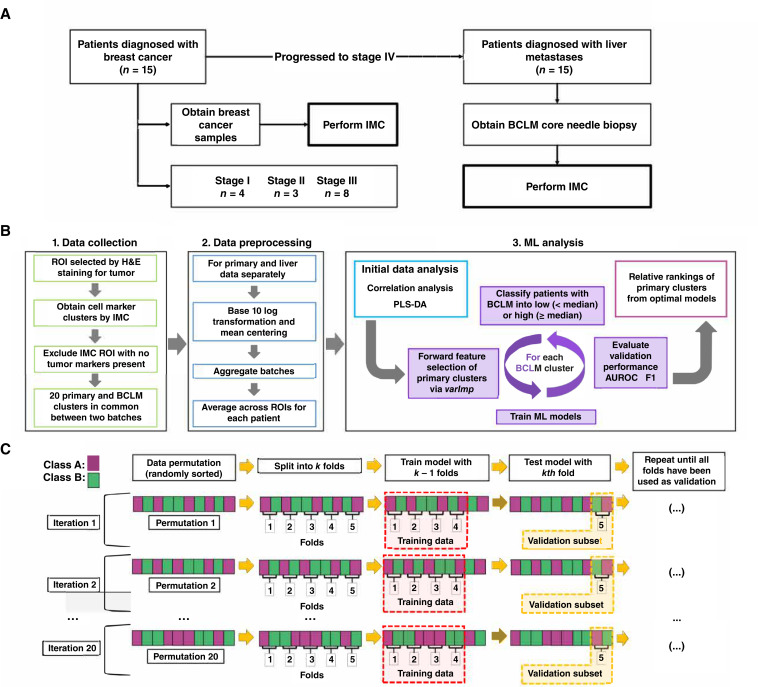
Workflow of study design. **A,** Study profile. Primary breast cancer samples were taken from 15 patients. After subsequent diagnosis of BCLM, a core needle biopsy was also obtained. **B,** Summary of analysis. ROIs from primary tumor and BCLM were identified using H&E staining for tumor tissue and TME. Multiple marker clusters were quantified by IMC, producing 20 common clusters between two analytical batches. IMC ROI missing multiple tumor markers (Ki-67, E-cad^+^, or αSMA) were excluded. Multiple ML models were trained to classify BCLM cluster expression into low (< median) or high (≥ median) groups using primary tumor cluster data. Forward feature selection was performed on preprocessed data using *varImp* to identify primary TME markers associated with BCLM classification. **C,** Diagram of model training and validation. Primary tumor data were randomly sorted and split into k folds (subsets; here, *k* = 5). Each model was trained with k-1 folds and validated with the kth fold. This process was repeated until all folds were used once as the validation set. Twenty permutations were performed in total, repeating the validation process for each fold within each permutation. Final results of each model are the averages of the validations across all folds and all iterations (*n* = 100). H&E, hematoxylin and eosin.

Characteristics related to the matched (primary breast tumor and BCLM) samples, as well as the number of IMC ROI samples preserved for analysis per patient, are summarized in Table 1. All patients were female. Average patient age was 49.2 years (SD 12.7 years) with BCLM core needle biopsy taken on average 2.5 years (SD 1.2 years) after primary tumor resection (by mastectomy). Most patients when initially diagnosed had American Joint Committee on Cancer (AJCC) stage III (*n* = 8), followed by stage I (*n* = 4) and stage II (*n* = 3). Most patients were T1 (*n* = 7) or T2 (*n* = 6), with only two being T3 (*n* = 1) or T4 (*n* = 1). Patients were roughly evenly distributed across lymph node classifications: N0 (*n* = 3), N1 (*n* = 4), N2 (*n* = 5), and N3 (*n* = 3). For most patients, primary tumor ER, PR, and HER2 classifications matched those in the metastasis. For HER2 staining, scores of 0 and 1+ were considered negative, score 2+ was borderline (qFISH information was unavailable from the vendor), and score 3+ was considered HER2-positive. Primary tumors included one triple-negative, seven HER2-negative (with ER- or PR-positive), five HER2 borderline (with two ER/PR-negative and three ER- or PR-positive), two HER2-positive (both ER/PR-negative), and no triple-positive. Although 10 patients had either ER^+^ or PR^+^ primary tumors (with nine of them also ER^+^ or PR^+^ BCLM), only one of the HER2-positive primary cases had HER2^+^ BCLM. The study methodology is summarized in Fig. 1.

**Table 1:** Characteristics of patients with breast cancer. TNM classification was 0 and 1 for primary tumor and liver metastatic disease, respectively

Patient	Disease status	Age at sample collection	Grade	Primary tumor (T) classification	Regional lymph node (N) classification	AJCC staging	ER intensity, % positivity	PR intensity, % positivity	HER2	#IMC ROI samples for ML analysis
1	Primary	55	II	1	2	IIIA	—	—	3+	5
	Metastatic	57	II	N/A	N/A	IV	—	—	3+	4
2	Primary	53	III	2	2	IIIA	—	—	0	5
	Metastatic	56	III	N/A	N/A	IV	—	—	0	5
3	Primary	63	R: II L: II	R: 2 L: 2	R: 1a L: 0	R: IIB L: IIA	—	—	2+	5
	Metastatic	64	N/A	N/A	N/A	IV	—	—	2+	4
4	Primary	35	III	3	3a	IIIC	Medium, 1%	—	0	5
	Metastatic	36	N/A	N/A	N/A	IV	—	—	0	4
5	Primary	51	III	1a	0	IA	—	—	3+	5
	Metastatic	53	N/A	N/A	N/A	IV	—	—	2+	2
6	Primary	30	II	1c	0	IA	Weak, 60%	Medium, 3%	2+	4
	Metastatic	32	N/A	N/A	N/A	IV	Medium, 6%	Medium, 2%	2+	2
7	Primary	51	III	2	2a	IIIA	Weak, 5%	Medium, 3%	1+	5
	Metastatic	52	N/A	N/A	N/A	IV	Medium, 15%	Medium, 50%	2+	2
8	Primary	59	III	4b	2a	IIIB	—	—	2+	4
	Metastatic	61	N/A	N/A	N/A	IV	—	—	2+	3
9	Primary	28	II	2	3	IIIC	Medium, 30%	—	0	5
	Metastatic	31	N/A	N/A	N/A	IV	Medium, 60%	Weak, 8%	1+	5
10	Primary	69	II	2	2	IIIA	Strong. 100%	Strong, 80%	1+	5
	Metastatic	73	N/A	N/A	N/A	IV	Strong, 95%	Strong, 65%	2+	5
11	Primary	49	R: III L: II	R: 2 L: 1c	R: 3 L: 1	R: IIIC L: IIA	R: Medium, 40% L: Medium, 70%	R: Strong, 70% L: Strong, 30%	R: 1+ L: 0	5
	Metastatic	53	N/A	N/A	N/A	IV	Strong, 95%	—	1+	3
12	Primary	45	II	1b	1mi	IB	Strong, 70%	Strong, 65%	1+	5
	Metastatic	48	N/A	N/A	N/A	IV	Weak, 5%	—	2+	5
13	Primary	32	N/A	1c	1	IIA	Strong, 90%	Strong, 90%	1+	5
	Metastatic	36	N/A	N/A	N/A	IV	Weak, 50%	—	2+	4
14	Primary	61	II	1c	0	IA	Weak, 50%	Strong, 40%	2+	5
	Metastatic	65	N/A	N/A	N/A	IV	Strong, 50%	Strong, 6%	2+	3
15	Primary	57	II	1c	1	IIA	Medium, 20%	—	2+	5
	Metastatic	58	N/A	N/A	N/A	IV	Medium, 30%	—	2+	4

Note: “1mi” denotes micrometastases (<2 mm), classified as 1. For HER2 staining, scores of 0 and 1+ are considered negative; score 2+ is borderline (qFISH information was unavailable from the vendor); and score 3+ is HER2-positive.

Abbreviations: N/A, not available; “R,” right; “L,” left; TNM, tumor–node–metastasis.

### Marker clusters identified from IMC data

**Figure 2:**
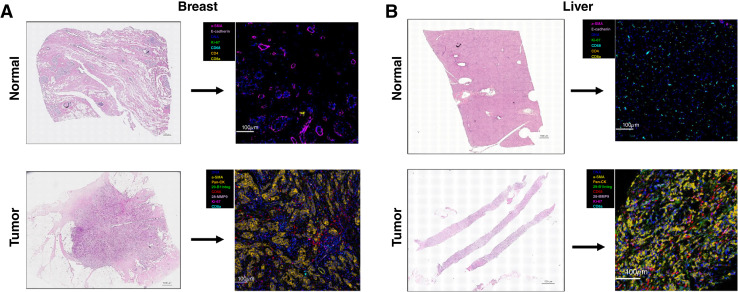
Representative H&E-stained slices of normal and tumor tissue, and corresponding IMC images from (**A**) breast and (**B**) liver tissue. H&E, hematoxylin and eosin.

**Figure 3:**
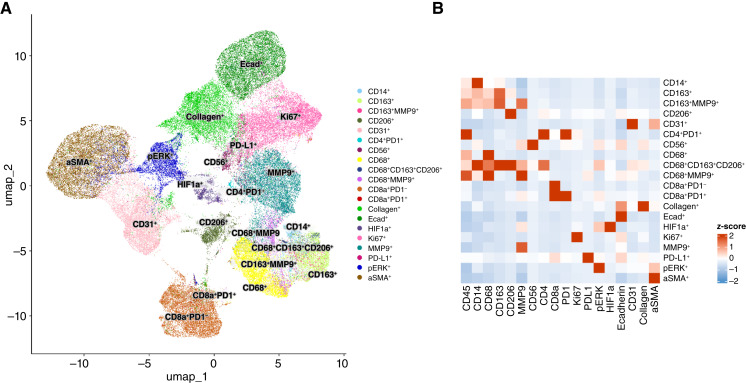
IMC raw data. **A,** UMAP representation of 20 identified clusters from breast primary and metastatic liver tumors. **B,** Heatmap of corresponding cluster and marker intensities from the IMC assay. UMAP, Uniform Manifold Approximation and Projection for Dimension Reduction.

Representative matched primary tumor and BCLM samples are shown in Fig. 2. The IMC cluster densities originating from primary tumor and BCLM before and after mean aggregation of ROIs are visualized in Supplementary Figs. S1 and S2, respectively. To confirm that the clusters were not skewed by analytical batch, a PLS-DA of the postpreprocessed data was performed, showing that the batches were homogeneous (Supplementary Fig. S3). Out of 23 marker clusters identified across batches, 20 were shared among all batches and were kept for analysis. Uniform Manifold Approximation and Projection (UMAP) representation of the 20 identified clusters from primary and metastatic liver tumors and a heatmap of the corresponding cluster and marker IMC intensities are shown in Fig. 3, whereas a representative example of mapping of the annotated phenotype to corresponding segmented cells based on the markers identified by IMC for paired primary tumor and BCLM is illustrated in Supplementary Fig. S4.

Primary tumor and BCLM IMC markers in preserved clusters (Table 2) were associated with macrophages (CD68, CD163, and CD206), monocytes (CD14), immune response (CD56, CD4, and CD8a), PD1, PD-L1, tumor tissue (Ki-67 and phosphorylated ERK), cell adhesion (E-cad), hypoxia (HIF1α), vascularity (CD31), and ECM (αSMA, collagen, and MMP9). To evaluate whether IMC clusters from patients with BCLM could in principle be separated into low (< median) or high (≥ median) groups using primary tumor cluster densities, a PLS-DA showed that this separation was feasible (*P* < 0.01; Supplementary Fig. S5). This separation was not possible using covariates only (Supplementary Fig. S6). A comparison of marker cluster densities of patients whose tumor subtypes differed between primary tumor and liver metastasis to those patients whose subtypes matched indicates that CD68^+^MMP9^+^ in the primary was lower (*P* = 0.034) in patients with differing ER subtype, whereas BCLM HIF1α^+^ and CD163^+^MMP9^+^ were lower (*P* = 0.014 and 0.032, respectively) and E-cad^+^ was higher (*P* = 0.018) in patients with differing HER2 subtype. There were no disparities based on differing PR status.

**Table 2:** Markers identified by IMC in primary breast tumor and BCLM samples

Biological marker	Associated cell expressions in TME	Metal/channels for IMC analysis	Antibody source for IMC (vendor/clone)	Reference
CD14	Monocytes/macrophages – co-receptor of Toll-like receptor 4	Gd160	Abcam/EPR3653	5
CD163	TAM, alternatively activated or anti-inflammatory macrophage (M2) scavenger receptor	Sm152	Bio-Rad/MCA1853	7
CD206	M2 macrophages – mannose receptor	Nd144	Abnova/22-130	39
CD31	Endothelial marker	Sm149	Abcam/C31.3+JC/70A	16
CD4	Helper T cells – transmembrane glycoprotein	Nd145	Abcam/EPR6855	22
CD56	NK mainly and some T cells, monocytes, and dendritic cells	Yb176	Invitrogen/123C3	23
CD68	Circulating/tissue macrophages (monocytic lineage), mainly M1 – promote phagocytosis	Tb159	BioLegend/ KP1	35
CD8a	Cytotoxic T cells and some NKs – transmembrane glycoprotein	Nd146	Invitrogen/C8/144B	55
Collagen	Collagen marker	Tm169	Novus/ Polyclonal	18
E-cad	Cell adhesion; expressed in normal breast tissue	Gd158	Cell Signaling Technology/24E10	50
HIF1α	Hypoxia	Ho165	Abcam/EP1215Y	17
Ki-67	Cancer cells – proliferation	Er167	BD Biosciences/B56	14
MMP9	Tissue remodeling and inflammation	Yb172	Abcam/ EP1255Y	20
PD1	Immune cells including T cells – programmed cell death protein 1	Eu151	BioLegend/NAT105	21
PD-L1	Suppression of adaptive immunity – cancer prognostic marker	Nd150	BioLegend/ 29E2A3	21
pERK	Signal transduction protein – regulates a variety of cellular processes	Sm147	Cell Signaling Technology/T202/Y204	15
αSMA	Alpha smooth muscle actin; marker for fibrogenic activity	Pr141	Invitrogen/1A4	19

### Correlations between marker clusters

**Figure 4:**
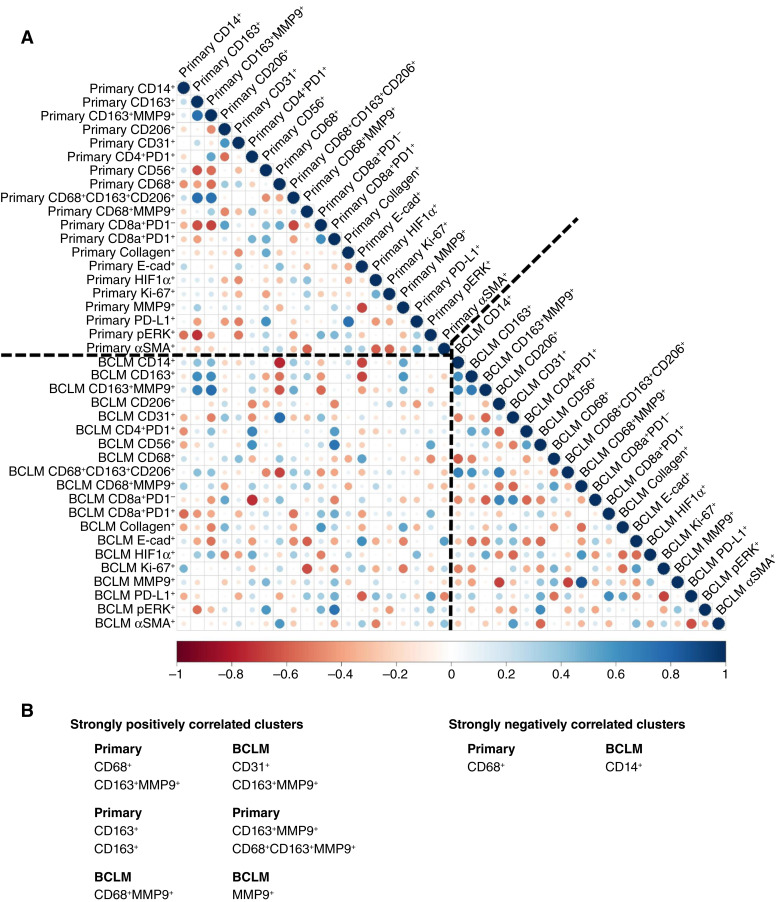
IMC cluster correlations. **A,** Correlations between primary tumor (breast) and BCLM (liver) marker clusters. **B,** Pairs of strongly correlated clusters (|*ρ*| ≥ 0.75).

To quantify the relationships between cluster densities, a correlation analysis between primary tumor and BCLM data was performed (Fig. 4). Within primary tumor, CD163^+^ was positively correlated with CD163^+^MMP9^+^ (*r* = 0.751, *P* = 0.0013) and CD68^+^CD163^+^CD206^+^ (*ρ* = 0.762, *P* = 9.7E−4). For BCLM, CD68^+^MMP9^+^ was positively correlated with MMP9^+^ (*r* = 0.861, *P* = 3.8E−5). Strong correlations between primary tumor clusters and BCLM clusters were observed that highlight relationships between the respective TMEs. Primary tumor CD68^+^ expression was negatively correlated with BCLM CD14^+^ (*r* = −0.751, *P* = 0.0012) and positively correlated with BCLM CD31^+^ (r = 0.763, *P* = 9.3E−4). Additionally, primary tumor CD163^+^MMP9^+^ was positively correlated with BCLM CD163^+^MMP9^+^ (*r* = 0.763, *P* = 9.4E−4). Interestingly, there were no strong correlations for HIF1α, Ki67, MMP9, or PD-L1 at the primary with marker clusters in the BCLM. Because no primary tumor clusters were found to significantly differ after Bonferroni adjustment between patients separated by low (< median) or high (≥ median) BCLM cluster expression (*P*-adj = 0.0025), statistical analyses alone were considered insufficient to predict the BCLM TME from the primary tumor TME.

### Prediction of BCLM TME from primary tumor marker clusters

**Figure 5:**
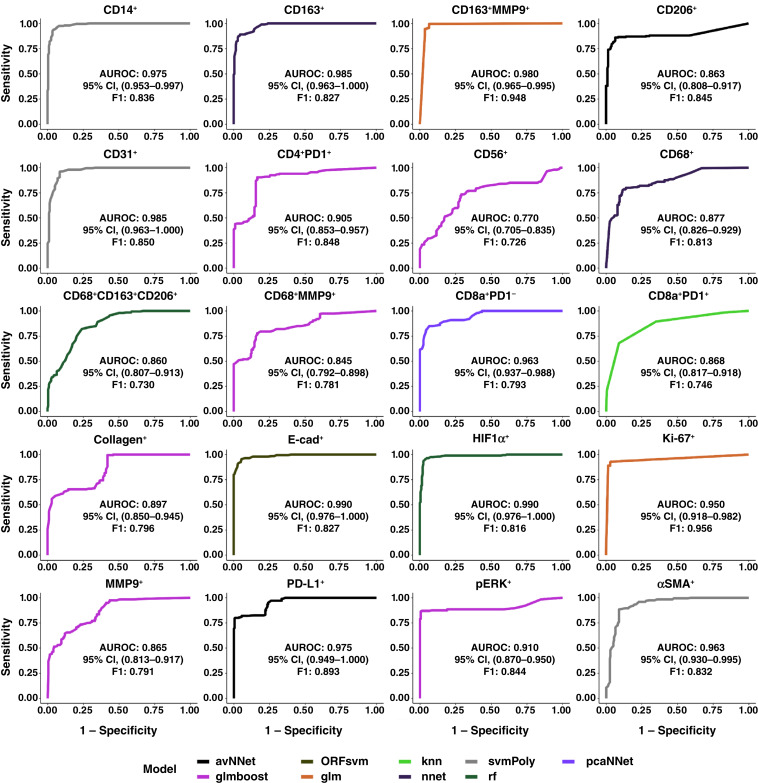
AUROC curves of ML models using primary IMC tumor cluster densities as input features to predict BCLM IMC cluster densities as being either above or below median values. One ML model was created per BCLM cluster.

To evaluate the predictive potential of the primary tumor TME at predicting the BCLM TME, ML models were trained to classify BCLM cluster densities as having low (< median) or high (≥ median) values. Using all 20 primary marker clusters, the models were selected by maximum validation subset AUROC per BCLM cluster (Supplementary Table S1). AUROC for all optimized models was ≥0.75, with 95% confidence in all cases ≥0.70 (Fig. 5). This performance was supported by F1 ≥ 0.70 for all models. The lowest AUROC and F1 were for predicting BCLM CD56^+^, with *glmboost* AUROC = 0.770 (95% CI, 0.705–0.835) and F1 = 0.726. Variable importance of primary tumor clusters to predict BCLM clusters is summarized in Supplementary Table S2 through Supplementary Table S21 and visualized in Supplementary Fig. S7 through Supplementary Fig. S11. AUROC and F1 across feature subsets are visualized in Supplementary Figs. S12 and S13, respectively.

### Prediction of BCLM TME using covariates

To evaluate the predictive value of covariates alone to predict the BCLM TME, the ML workflow was executed using covariates as features. Covariates included patient age at primary tumor resection, time between primary tumor and BCLM sample collections, T and N components from tumor–node–metastasis score, AJCC staging, and cell receptor status. Patients with more than one tumor–node–metastasis, AJCC, or receptor level were ascribed the more advanced level. Only CD68^+^CD163^+^CD206^+^ had higher validation subset AUROC using covariates (0.922; 95% CI, 0.890–0.955) than cluster density–informed ML models (0.860; 95% CI, 0.807–0.913; Supplementary Figu. S14). Consistent with the PLS-DA classification using covariates only (Supplementary Fig. S6), these results indicate that covariates alone would generally underperform to predict the BCLM TME compared with the marker clusters.

### Identification of key primary tumor marker clusters

**Figure 6:**
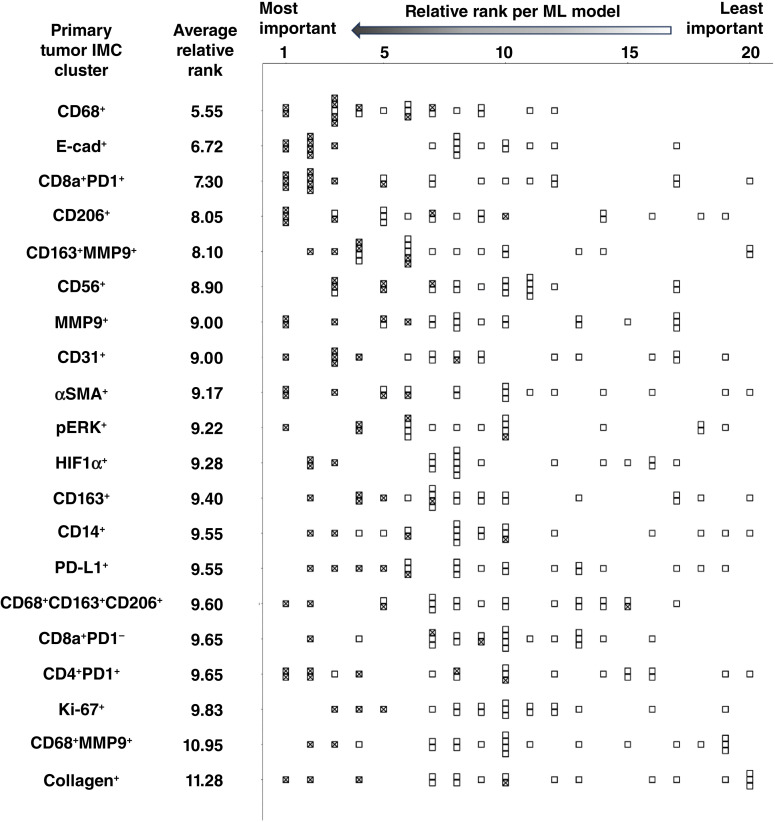
Primary tumor IMC clusters sorted by average relative rank across the ML models (lowest number denotes the highest rank) based on their importance for classification of the BCLM IMC clusters. Each box denotes the relative rank of a cluster in a particular model (with lowest rank as most important), whereas a checked box highlights the relative rank of a cluster when included by a model for prediction of BCLM cluster densities.

To identify which primary tumor marker clusters were most important to predict the BCLM TME, the variable importance rank for each primary tumor cluster was determined for prediction of each of the 20 BCLM cluster densities. Comparisons between differing ML feature rankings show that CD68^+^ had the highest average relative rank of all 20 clusters (5.55), whereas collagen^+^ had the lowest (11.28; Fig. 6).

## Discussion

This study provides evidence that the BCLM TME can in principle be inferred from the primary breast tumor tissue. Using a dataset of patient-matched primary breast cancer and BCLM samples, IMC cluster densities from BCLM were predicted as being either above or below median values using IMC cluster density data from the primary tumor (AUROC ≥ 0.75 on the validation subset with 95% CI ≥ 0.7 for all markers). Because tissue from primary tumors is usually obtained (e.g., via resection), the proposed approach involving IMC analysis of primary tumor tissue and ML could longer term aid in targeting BCLM with immunotherapeutic regimens without requiring BCLM tissue analysis, which is generally unavailable. These regimens critically depend on the characteristics of the associated TME, such as tumor-associated macrophages (TAM). The TME has an important role not only in immunotherapy but also in other therapeutic approaches, emphasizing the need for patient-specific evaluation of liver metastases to optimize treatment efficacy.

Rankings of different primary tumor clusters based on their importance for ML classification highlight the predictive value of macrophages. These rankings indicate that primary tumor CD68, a pan-macrophage marker (35), was the most significant feature overall in the primary TME for predicting the BCLM TME (Fig. 6). Interestingly, CD31 expression in BCLM was positively correlated with CD68^+^ in the primary (Fig. 4B), suggesting that vascularization at the metastatic site was associated with macrophage presence at the primary. Furthermore, CD163, a marker of TAMs, especially M2, was present in CD163^+^MMP9^+^ and CD163^+^ primary tumor clusters (Fig. 6; ref. 7). The marker cluster data also indicate that BCLM CD163^+^ coupled with MMP9^+^ was positively correlated with the same at the primary (Fig. 4B), indicating that primary tumor intrinsic characteristics were reflected at the remote site. MMPs are well known as key components of the tumor-promoting and immune-suppressive TME (36). In contrast, monocyte/macrophage presence (CD14^+^) in BCLM was negatively correlated with M1 (CD68^+^) macrophage presence in the primary (Fig. 4B), suggesting that macrophage involvement at the metastatic site is linked to the extent of M1 antitumor activity at the primary. Interestingly, the comparison of marker cluster densities of patients whose tumor subtypes differed between primary and BCLM to those patients with consistent subtypes revealed that CD68^+^MMP9^+^ in the primary was lower in patients with differing ER subtype whereas BCLM CD163^+^MMP9^+^ and HIF1α^+^ were lower in patients with differing HER2 subtype. ER^+^ tumors have been shown to exhibit differential macrophage phenotypes (37), whereas a prognostic role for TAMs in HER2^+^ tumors has been established (38). Additionally, CD206 expressed primarily by M2-like macrophages and dendritic cells (39) was the fourth most important feature by an average relative rank (Fig. 6).

These findings are consistent with previous work documenting macrophage influence on cancer progression and that the M1:M2 ratio can serve as a prognostic marker in multiple tumor types when considering conventional therapies (40). M1 macrophages target tumor cells via multiple mechanisms, including release of inflammatory cytokines (such as IL6 and TNFα), reactive oxygen species, nitrogen intermediates, and other factors, whereas M2 promotes immunosuppression, tumor growth, progression, and resistance to therapy via regulation of factors such as TGFβ and IL10, VEGF-A that mediates angiogenesis, and other growth factors (41). TGFβ has been linked to ECM dysregulation across multiple cancer types (42). Our previous work has found that M2 macrophages could potentially sensitize tumors to nanotherapy-mediated drug delivery (11). Furthermore, macrophages influence T-cell activation and function (43). Altogether, based on the multifaceted influence of macrophages in the TME, the results in this study could help in identifying patients who may benefit from T cell–based immunotherapies, especially as macrophages are known to limit cytotoxic T-cell efficacy in liver metastases (44).

The CD8a^+^PD1^+^ cluster (Fig. 6) combines CD8a, a cytotoxic T-cell surface marker, and PD1, a CD8^+^ T-cell marker for which increased and sustained expression is associated with PD-L1–induced T-cell exhaustion (Table 2). Exhausted cytotoxic T cells characterize decreased immunotherapeutic efficacy in breast cancer (45). Inhibiting PD-L1 has been shown to improve survival in triple-negative breast cancer independently of pathologic complete response (46). Interestingly, it has been reported that PD-L1 expression in liver metastasis of colorectal tumors was higher than that in primary tumors (47). Future work could explore the potential of therapy targeting PD1 or PD-L1 expression combined with nanovector-mediated tumor cytotoxic drug delivery. This would require evaluating the levels of PD1 expression, which were not assessed in this study’s IMC, to determine whether the cytotoxic T-cell population found on primary tumors corresponds to exhausted CD8^+^ T cells in the BCLM. However, preclinical models have found that resistance to anti-PD1/PD-L1 antibody therapy can be explained by the immunotolerant microenvironment of the liver (48). It is, therefore, unclear whether predicting PD-L1 expression in the liver metastases could translate into effective immunotherapy combinations for breast or other cancer types. Lastly, the CD56^+^ primary tumor cluster indicates NK cell presence, which may reflect sensitivity to NK-mediated cytotoxicity (49).

In addition to the importance of markers associated with immune cells in the primary tumor to predict the BCLM TME, the relevance of primary tumor cell–ECM interactions, including E-cad, MMP9, and αSMA, was evident by their rankings for ML classification (Fig. 6). Previous work has shown that E-cad expression is required for metastatic formation, whereas low E-cad may increase tumor invasiveness; recently, the functional state of E-cad was shown to determine metastatic potential in a preclinical model of breast cancer (50). MMP9 expression has been consistently associated with tumor growth and metastasis and has been considered a potential therapeutic target in breast cancer (20).

Despite the wide range of markers covered by the clusters detected in this study, these markers are only associated with a subset of the TME. T-cell markers such as forkhead box protein P3 (51) or CD45 (52) could be included to further assess T cell–mediated activity. The influence of other immune markers, such as CD86 expressed by B cells and antigen presenting cells (53), could be investigated. Although the availability of matched human primary and BCLM samples is limited and only 15 pairs were available for this proof-of-concept study, future work will strive for larger sample sizes and diverse sampling pools to prospectively confirm and further refine the ML model predictions. BCLM ROIs were obtained via core needle biopsies, which, in the case of metastatic malignancies in the liver, have been found to afford more tissue for analysis and improve diagnostic quality relative to fine needle aspiration biopsies (54). Considering the rarity of matched breast and BCLM samples, this study emphasized cohort size; thus, BCLM ROI per patient was varied from two to five depending on the number of ROI deemed eligible for analysis. Subsequent studies could focus on additional ROI to overcome this limitation. Although tissue-specific sample details, IHC, and tissue staging details are known, no details were available from the vendor with regard to treatment regimens pre-biopsy, after initial breast cancer diagnosis, or after liver metastasis diagnosis. The effect of therapy on primary and metastatic TMEs will need future evaluation. In addition to information obtained from primary tumor biopsies and resections, other predictors of the BCLM TME such as from proteomic or metabolomic analysis could be investigated. Mechanistic modeling using primary tumor characteristics could be used to predict BCLM response to immunotherapy, e.g., by applying techniques that have simulated BCLM response to nanotherapy targeting TAMs (9). A combination of such approaches could help bolster the TME links between primary breast tumors and BCLM to arrive at optimal patient-specific immunotherapeutic strategies.

## Supplementary Material

Supplementary Figure S1 S1. Heatmap of IMC cluster densities originating from breast primary and breast cancer liver metastases (BCLM) before mean aggregation of ROIs.

Supplementary Figure S2 S2. Heatmap of IMC cluster densities originating from BCLM (top) and primary breast tumors (bottom) after mean aggregation of ROIs.

Supplementary Figure S3 S3. PLS-DA of primary breast and BCLM IMC ROI data by batch number showing that the batches were homogeneous.

Supplementary Figure S4 S4. Example of mapping of the annotated phenotype to corresponding segmented cells based on the markers identified by IMC for paired primary tumor and BCLM (patient 15).

Supplementary Figure S5 S5. PLS-DA score plots of classifying BCLM IMC cluster densities into Low (<median) or High (≥median) groups using primary tumor IMC cluster densities.

Supplementary Figure S6 S6. PLS-DA score plots of classifying BCLM patient IMC clusters into Low (<median) or High (≥median) groups using covariates only.

Supplementary Figure S7 S7. Variable importance for prediction of BCLM CD14+, CD163+, CD163+MMP9+, and CD206+ using ML models.

Supplementary Figure S8. S8. Variable importance for prediction of BCLM CD31+, CD4+PD1+, CD56+, and CD68+ using ML models.

Supplementary Figure S9 S9. Variable importance for prediction of BCLM CD68+CD163+CD206+, CD68+MMP9+, CD8a+PD1-, and CD8a+PD1+ using ML models.

Supplementary Figure S10 S10. Variable importance for prediction of BCLM Collagen+, E-cad+, HIF1α+, and Ki-67+ using ML models.

Supplementary Figure S11 Table S11. Variable importance for prediction of BCLM MMP9+, PD-L1+, pERK+, and αSMA+ using ML models.

Supplementary Figure S12 S12. AUROC achieved by ML models using primary tumor IMC clusters across a variable number of features to predict BCLM IMC cluster densities (as stated in gray box of each panel)

Supplementary Figure S13 S13. Metric F1 achieved by ML models using primary tumor IMC clusters across a variable number of features to predict BCLM IMC cluster densities (as stated in gray box of each panel)

Supplementary Figure S14 S14. AUROC curves of ML models using covariates only to predict BCLM IMC cluster densities as being either above or below median values.

Supplementary Table S1 Table S1. Hyperparameters used for AUROC-optimized Machine Learning models.

Supplementary Table S2 Table S2. Variable Importance for predicting BCLM CD14+ using primary tumor clusters.

Supplementary Table S3 Table S3. Variable Importance for predicting BCLM CD163+ using primary tumor clusters.

Supplementary Table S4 Table S4. Variable Importance for predicting BCLM CD163+MMP9+ using primary tumor clusters.

Supplementary Table S5 Table S5. Variable Importance for predicting BCLM CD206+ using primary tumor clusters.

Supplementary Table S6 Table S6. Variable Importance for predicting BCLM CD31+ using primary tumor clusters.

Supplementary Table S7 Table S7. Variable Importance for predicting BCLM CD4+PD1+ using primary tumor clusters.

Supplementary Table S8 Table S8. Variable Importance for predicting BCLM CD56+ using primary tumor clusters.

Supplementary Table S9 Table S9. Variable Importance for predicting BCLM CD68+ using primary tumor clusters.

Supplementary Table S10 Table S10. Variable Importance for predicting BCLM CD68+CD163+CD206+ using primary tumor clusters.

Supplementary Table S11 Supplementary Table S11

Supplementary Table S12 Table S12. Variable Importance for predicting BCLM CD8a+PD1- using primary tumor clusters.

Supplementary Table S13 Table S13. Variable Importance for predicting BCLM CD8a+PD1+ using primary tumor clusters.

Supplementary Table S14 Table S14. Variable Importance for predicting BCLM Collagen+ using primary tumor clusters.

Supplementary Table S15 Table S15. Variable Importance for predicting BCLM E-cad+ using primary tumor clusters.

Supplementary Table S16 Supplementary Table S16. Variable Importance for predicting BCLM HIF1α+ using primary tumor clusters.

Supplementary Table S17 Table S17. Variable Importance for predicting BCLM Ki-67+ using primary tumor clusters.

Supplementary Table S18 Table S18. Variable Importance for predicting BCLM MMP9+ using primary tumor clusters.

Supplementary Table S19 Table S19. Variable Importance for predicting BCLM PD-L1+ using primary tumor clusters.

Supplementary Table S20 Table S20. Variable Importance for predicting BCLM pERK+ using primary tumor clusters.

Supplementary Table S21 Supplementary Table S21. Variable Importance for predicting BCLM αSMA+ using primary tumor clusters.

## References

[1] Zhao H-Y , Gong Y, Ye F-G, Ling H, Hu X. Incidence and prognostic factors of patients with synchronous liver metastases upon initial diagnosis of breast cancer: a population-based study. Cancer Manag Res 2018;10:5937–50. 30538544 10.2147/CMAR.S178395 PMC6255056

[2] Sheafor DH , Frederick MG, Paulson EK, Keogan MT, DeLong DM, Nelson RC. Comparison of unenhanced, hepatic arterial-dominant, and portal venous-dominant phase helical CT for the detection of liver metastases in women with breast carcinoma. AJR Am J Roentgenol 1999;172:961–8. 10587129 10.2214/ajr.172.4.10587129

[3] Frieboes HB , Raghavan S, Godin B. Modeling of nanotherapy response as a function of the tumor microenvironment: focus on liver metastasis. Front Bioeng Biotechnol 2020;8:1011. 32974325 10.3389/fbioe.2020.01011 PMC7466654

[4] Cheng K , Cai N, Zhu J, Yang X, Liang H, Zhang W. Tumor-associated macrophages in liver cancer: from mechanisms to therapy. Cancer Commun (Lond) 2022;42:1112–40. 36069342 10.1002/cac2.12345 PMC9648394

[5] Ginhoux F , Jung S. Monocytes and macrophages: developmental pathways and tissue homeostasis. Nat Rev Immunol 2014;14:392–404. 24854589 10.1038/nri3671

[6] Gomez Perdiguero E , Klapproth K, Schulz C, Busch K, Azzoni E, Crozet L, . Tissue-resident macrophages originate from yolk-sac-derived erythro-myeloid progenitors. Nature 2015;518:547–51. 25470051 10.1038/nature13989 PMC5997177

[7] Allison E , Edirimanne S, Matthews J, Fuller SJ. Breast cancer survival outcomes and tumor-associated macrophage markers: a systematic review and meta-analysis. Oncol Ther 2023;11:27–48. 36484945 10.1007/s40487-022-00214-3 PMC9935786

[8] Tanei T , Leonard F, Liu X, Alexander JF, Saito Y, Ferrari M, . Redirecting transport of nanoparticle albumin-bound paclitaxel to macrophages enhances therapeutic efficacy against liver metastases. Cancer Res 2016;76:429–39. 26744528 10.1158/0008-5472.CAN-15-1576 PMC4715951

[9] Leonard F , Curtis LT, Yesantharao P, Tanei T, Alexander JF, Wu M, . Enhanced performance of macrophage-encapsulated nanoparticle albumin-bound-paclitaxel in hypo-perfused cancer lesions. Nanoscale 2016;8:12544–52. 26818212 10.1039/c5nr07796f PMC4919151

[10] Leonard F , Curtis LT, Ware MJ, Nosrat T, Liu X, Yokoi K, . Macrophage polarization contributes to the anti-tumoral efficacy of mesoporous nanovectors loaded with albumin-bound paclitaxel. Front Immunol 2017;8:693. 28670313 10.3389/fimmu.2017.00693 PMC5472662

[11] Leonard F , Curtis LT, Hamed AR, Zhang C, Chau E, Sieving D, . Nonlinear response to cancer nanotherapy due to macrophage interactions revealed by mathematical modeling and evaluated in a murine model via CRISPR-modulated macrophage polarization. Cancer Immunol Immunother 2020;69:731–44. 32036448 10.1007/s00262-020-02504-z PMC7186159

[12] Goodin DA , Chau E, Tiwari A, Godin B, Frieboes HB. Multiple breast cancer liver metastases response to macrophage-delivered nanotherapy evaluated via a 3D continuum model. Immunology 2023;169:132–40. 36465031 10.1111/imm.13615

[13] Stessels F , Van den Eynden G, Van der Auwera I, Salgado R, Van den Heuvel E, Harris AL, . Breast adenocarcinoma liver metastases, in contrast to colorectal cancer liver metastases, display a non-angiogenic growth pattern that preserves the stroma and lacks hypoxia. Br J Cancer 2004;90:1429–36. 15054467 10.1038/sj.bjc.6601727 PMC2409675

[14] Zhang A , Wang X, Fan C, Mao X. The role of Ki67 in evaluating neoadjuvant endocrine therapy of hormone receptor-positive breast cancer. Front Endocrinol (Lausanne) 2021;12:687244. 34803903 10.3389/fendo.2021.687244 PMC8597938

[15] Bartholomeusz C , Gonzalez-Angulo AM, Liu P, Hayashi N, Lluch A, Ferrer-Lozano J, . High ERK protein expression levels correlate with shorter survival in triple-negative breast cancer patients. Oncologist 2012;17:766–74. 22584435 10.1634/theoncologist.2011-0377 PMC3380875

[16] Schlüter A , Weller P, Kanaan O, Nel I, Heusgen L, Höing B, . CD31 and VEGF are prognostic biomarkers in early-stage, but not in late-stage, laryngeal squamous cell carcinoma. BMC Cancer 2018;18:272. 29523110 10.1186/s12885-018-4180-5 PMC5845191

[17] Bai R , Li Y, Jian L, Yang Y, Zhao L, Wei M. The hypoxia-driven crosstalk between tumor and tumor-associated macrophages: mechanisms and clinical treatment strategies. Mol Cancer 2022;21:177. 36071472 10.1186/s12943-022-01645-2 PMC9454207

[18] Aumailley M , Gayraud B. Structure and biological activity of the extracellular matrix. J Mol Med (Berl) 1998;76:253–65. 9535559 10.1007/s001090050215

[19] Muchlińska A , Nagel A, Popęda M, Szade J, Niemira M, Zieliński J, . Alpha-smooth muscle actin-positive cancer-associated fibroblasts secreting osteopontin promote growth of luminal breast cancer. Cell Mol Biol Lett 2022;27:45. 35690734 10.1186/s11658-022-00351-7 PMC9188043

[20] Juric V , O’Sullivan C, Stefanutti E, Kovalenko M, Greenstein A, Barry-Hamilton V, . MMP-9 inhibition promotes anti-tumor immunity through disruption of biochemical and physical barriers to T-cell trafficking to tumors. PLoS One 2018;13:e0207255. 30500835 10.1371/journal.pone.0207255 PMC6267998

[21] Zhang H , Liu L, Liu J, Dang P, Hu S, Yuan W, . Roles of tumor-associated macrophages in anti-PD-1/PD-L1 immunotherapy for solid cancers. Mol Cancer 2023;22:58. 36941614 10.1186/s12943-023-01725-x PMC10029244

[22] Boieri M , Malishkevich A, Guennoun R, Marchese E, Kroon S, Trerice KE, . CD4^+^ T helper 2 cells suppress breast cancer by inducing terminal differentiation. J Exp Med 2022;219:e20201963. 35657353 10.1084/jem.20201963 PMC9170526

[23] Vivier E , Ugolini S, Blaise D, Chabannon C, Brossay L. Targeting natural killer cells and natural killer T cells in cancer. Nat Rev Immunol 2012;12:239–52. 22437937 10.1038/nri3174 PMC5161343

[24] Botteri E , Disalvatore D, Curigliano G, Brollo J, Bagnardi V, Viale G, . Biopsy of liver metastasis for women with breast cancer: impact on survival. Breast 2012;21:284–8. 22212746 10.1016/j.breast.2011.12.014

[25] Yamamoto M , Yoshida M, Furuse J, Sano K, Ohtsuka M, Yamashita S, . Clinical practice guidelines for the management of liver metastases from extrahepatic primary cancers 2021. J Hepatobiliary Pancreat Sci 2021;28:1–25. 33200538 10.1002/jhbp.868

[26] Barba D , León-Sosa A, Lugo P, Suquillo D, Torres F, Surre F, . Breast cancer, screening and diagnostic tools: all you need to know. Crit Rev Oncol Hematol 2021;157:103174. 33249359 10.1016/j.critrevonc.2020.103174

[27] He Y , Han Y, Fan Ah, Li D, Wang B, Ji K, . Multi-perspective comparison of the immune microenvironment of primary colorectal cancer and liver metastases. J Transl Med 2022;20:454. 36195882 10.1186/s12967-022-03667-2 PMC9533561

[28] Julious SA . Sample size of 12 per group rule of thumb for a pilot study. Pharm Stat 2005;4:287–91.

[29] Berg S , Kutra D, Kroeger T, Straehle CN, Kausler BX, Haubold C, . ilastik: interactive machine learning for (bio)image analysis. Nat Methods 2019;16:1226–32. 31570887 10.1038/s41592-019-0582-9

[30] Stirling DR , Swain-Bowden MJ, Lucas AM, Carpenter AE, Cimini BA, Goodman A. CellProfiler 4: improvements in speed, utility and usability. BMC Bioinformatics 2021;22:433. 34507520 10.1186/s12859-021-04344-9 PMC8431850

[31] Schapiro D , Jackson HW, Raghuraman S, Fischer JR, Zanotelli VRT, Schulz D, . histoCAT: analysis of cell phenotypes and interactions in multiplex image cytometry data. Nat Methods 2017;14:873–6. 28783155 10.1038/nmeth.4391 PMC5617107

[32] Hao Y , Hao S, Andersen-Nissen E, Mauck WM 3rd, Zheng S, Butler A, . Integrated analysis of multimodal single-cell data. Cell 2021;184:3573–87.e29. 34062119 10.1016/j.cell.2021.04.048 PMC8238499

[33] Chung AW , Anand K, Anselme AC, Chan AA, Gupta N, Venta LA, . A phase 1/2 clinical trial of the nitric oxide synthase inhibitor L-NMMA and taxane for treating chemoresistant triple-negative breast cancer. Sci Transl Med 2021;13:eabj5070. 34910551 10.1126/scitranslmed.abj5070

[34] van den Berg RA , Hoefsloot HCJ, Westerhuis JA, Smilde AK, van der Werf MJ. Centering, scaling, and transformations: improving the biological information content of metabolomics data. BMC Genomics 2006;7:142. 16762068 10.1186/1471-2164-7-142 PMC1534033

[35] Jamiyan T , Kuroda H, Yamaguchi R, Abe A, Hayashi M. CD68- and CD163-positive tumor-associated macrophages in triple negative cancer of the breast. Virchows Arch 2020;477:767–75. 32607685 10.1007/s00428-020-02855-z PMC7683466

[36] Kessenbrock K , Plaks V, Werb Z. Matrix metalloproteinases: regulators of the tumor microenvironment. Cell 2010;141:52–67. 20371345 10.1016/j.cell.2010.03.015 PMC2862057

[37] Estrogen receptor-positive breast cancer subtypes show differential macrophage functions. Nat Cancer 2023;4:450–1. 36944697 10.1038/s43018-023-00528-9

[38] Honkanen TJ , Tikkanen A, Karihtala P, Mäkinen M, Väyrynen JP, Koivunen JP. Prognostic and predictive role of tumour-associated macrophages in HER2 positive breast cancer. Sci Rep 2019;9:10961. 31358801 10.1038/s41598-019-47375-2 PMC6662906

[39] Azad AK , Rajaram MVS, Schlesinger LS. Exploitation of the macrophage mannose receptor (CD206) in infectious disease diagnostics and therapeutics. J Cytol Mol Biol 2014;1:1000003. 24672807 10.13188/2325-4653.1000003 PMC3963702

[40] Komohara Y , Jinushi M, Takeya M. Clinical significance of macrophage heterogeneity in human malignant tumors. Cancer Sci 2014;105:1–8. 24168081 10.1111/cas.12314 PMC4317877

[41] Mantovani A , Allavena P, Marchesi F, Garlanda C. Macrophages as tools and targets in cancer therapy. Nat Rev Drug Discov 2022;21:799–820. 35974096 10.1038/s41573-022-00520-5 PMC9380983

[42] Chakravarthy A , Khan L, Bensler NP, Bose P, De Carvalho DD. TGF-β-associated extracellular matrix genes link cancer-associated fibroblasts to immune evasion and immunotherapy failure. Nat Commun 2018;9:4692. 30410077 10.1038/s41467-018-06654-8 PMC6224529

[43] Guerriero JL . Macrophages: their untold story in T cell activation and function. Int Rev Cell Mol Biol 2019;342:73–93. 30635094 10.1016/bs.ircmb.2018.07.001

[44] Yu J , Green MD, Li S, Sun Y, Journey SN, Choi JE, . Liver metastasis restrains immunotherapy efficacy via macrophage-mediated T cell elimination. Nat Med 2021;27:152–64. 33398162 10.1038/s41591-020-1131-x PMC8095049

[45] Tietscher S , Wagner J, Anzeneder T, Langwieder C, Rees M, Sobottka B, . A comprehensive single-cell map of T cell exhaustion-associated immune environments in human breast cancer. Nat Commun 2023;14:98. 36609566 10.1038/s41467-022-35238-w PMC9822999

[46] Loibl S , Schneeweiss A, Huober J, Braun M, Rey J, Blohmer J-U, . Neoadjuvant durvalumab improves survival in early triple-negative breast cancer independent of pathological complete response. Ann Oncol 2022;33:1149–58. 35961599 10.1016/j.annonc.2022.07.1940

[47] Wei X-L , Luo X, Sheng H, Wang Y, Chen D-L, Li J-N, . PD-L1 expression in liver metastasis: its clinical significance and discordance with primary tumor in colorectal cancer. J Transl Med 2020;18:475. 33308232 10.1186/s12967-020-02636-x PMC7730753

[48] Manfredi GF , Celsa C, John C, Jones C, Acuti N, Scheiner B, . Mechanisms of resistance to immunotherapy in hepatocellular carcinoma. J Hepatocell Carcinoma 2023;10:1955–71. 37941812 10.2147/JHC.S291553 PMC10629523

[49] Taouk G , Hussein O, Zekak M, Abouelghar A, Al-Sarraj Y, Abdelalim EM, . CD56 expression in breast cancer induces sensitivity to natural killer-mediated cytotoxicity by enhancing the formation of cytotoxic immunological synapse. Sci Rep 2019;9:8756. 31217484 10.1038/s41598-019-45377-8 PMC6584531

[50] Na T-Y , Schecterson L, Mendonsa AM, Gumbiner BM. The functional activity of E-cadherin controls tumor cell metastasis at multiple steps. Proc Natl Acad Sci U S A 2020;117:5931–7. 32127478 10.1073/pnas.1918167117 PMC7084067

[51] Lu L , Barbi J, Pan F. The regulation of immune tolerance by FOXP3. Nat Rev Immunol 2017;17:703–17. 28757603 10.1038/nri.2017.75 PMC5793224

[52] Rheinländer A , Schraven B, Bommhardt U. CD45 in human physiology and clinical medicine. Immunol Lett 2018;196:22–32. 29366662 10.1016/j.imlet.2018.01.009

[53] Collins M , Ling V, Carreno BM. The B7 family of immune-regulatory ligands. Genome Biol 2005;6:223. 15960813 10.1186/gb-2005-6-6-223 PMC1175965

[54] Suo L , Chang R, Padmanabhan V, Jain S. For diagnosis of liver masses, fine-needle aspiration versus needle core biopsy: which is better? J Am Soc Cytopathol 2018;7:46–9. 31043250 10.1016/j.jasc.2017.09.004

[55] Planes-Laine G , Rochigneux P, Bertucci F, Chrétien A-S, Viens P, Sabatier R, . PD-1/PD-L1 targeting in breast cancer: the first clinical evidences are emerging. A literature review. Cancers (Basel) 2019;11:1033. 31336685 10.3390/cancers11071033 PMC6679223

