# Supplementary material for: Characterization of the Breast Cancer Liver Metastasis Microenvironment via Machine Learning Analysis of the Primary Tumor Microenvironment
Source: Cancer Res Commun. 2024 Oct 31;4(10):2846–57. doi: 10.1158/2767-9764.CRC-24-0263 (PMC11525956; doi:10.1158/2767-9764.CRC-24-0263)
Supplement: Supplementary Figure S2 — S2. Heatmap of IMC cluster densities originating from BCLM (top) and primary breast tumors (bottom) after mean aggregation of ROIs. [file crc-24-0263_supplementary_figure_s2_suppsf2.pdf]

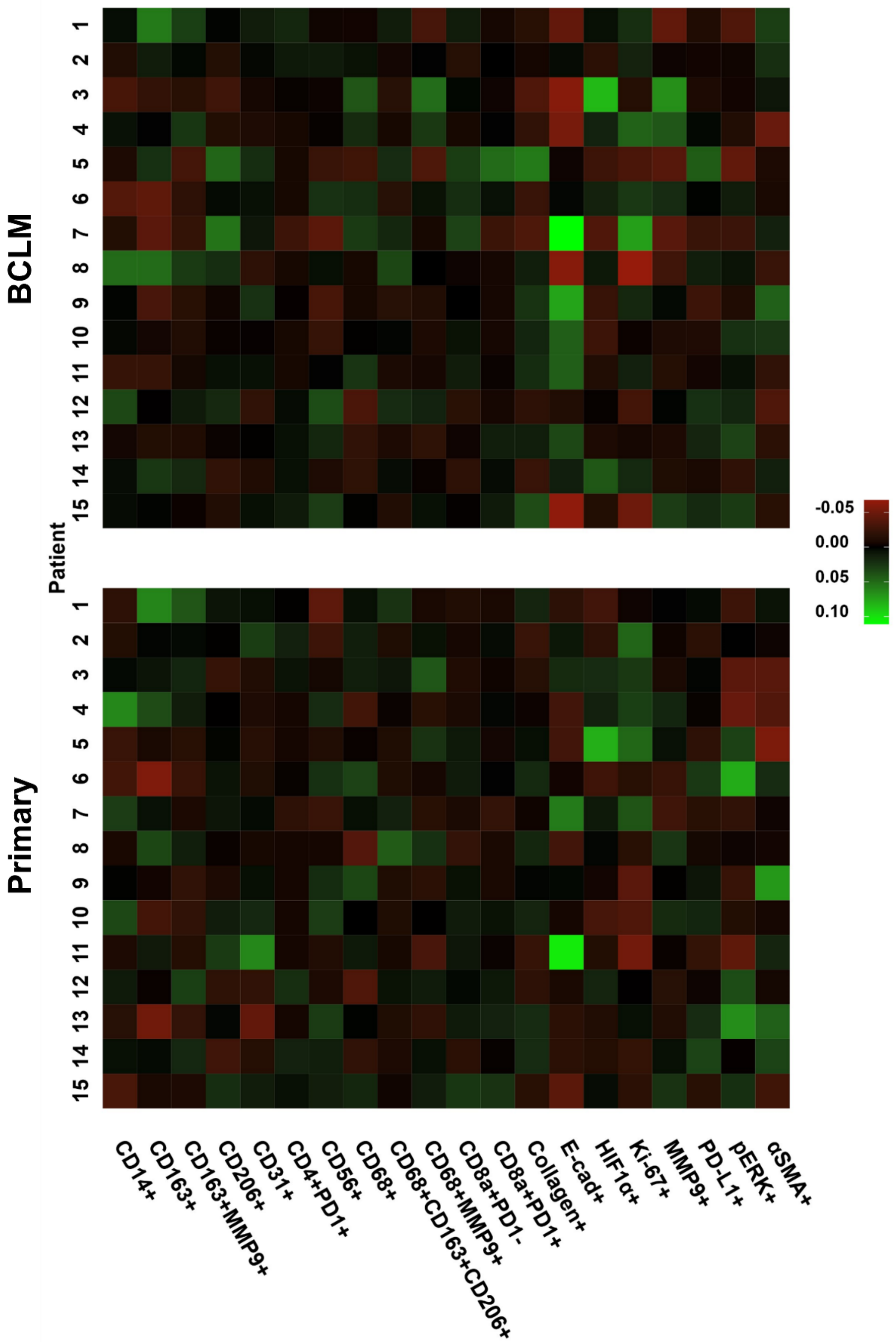

Supplementary Figure 2 – Heatmap of IMC cluster densities originating from BCLM (top) and primary breast tumors (bottom) after mean aggregation of ROIs.
