# Supplementary material for: Characterization of the Breast Cancer Liver Metastasis Microenvironment via Machine Learning Analysis of the Primary Tumor Microenvironment
Source: Cancer Res Commun. 2024 Oct 31;4(10):2846–57. doi: 10.1158/2767-9764.CRC-24-0263 (PMC11525956; doi:10.1158/2767-9764.CRC-24-0263)
Supplement: Supplementary Figure S3 — S3. PLS-DA of primary breast and BCLM IMC ROI data by batch number showing that the batches were homogeneous. [file crc-24-0263_supplementary_figure_s3_suppsf3.pdf]

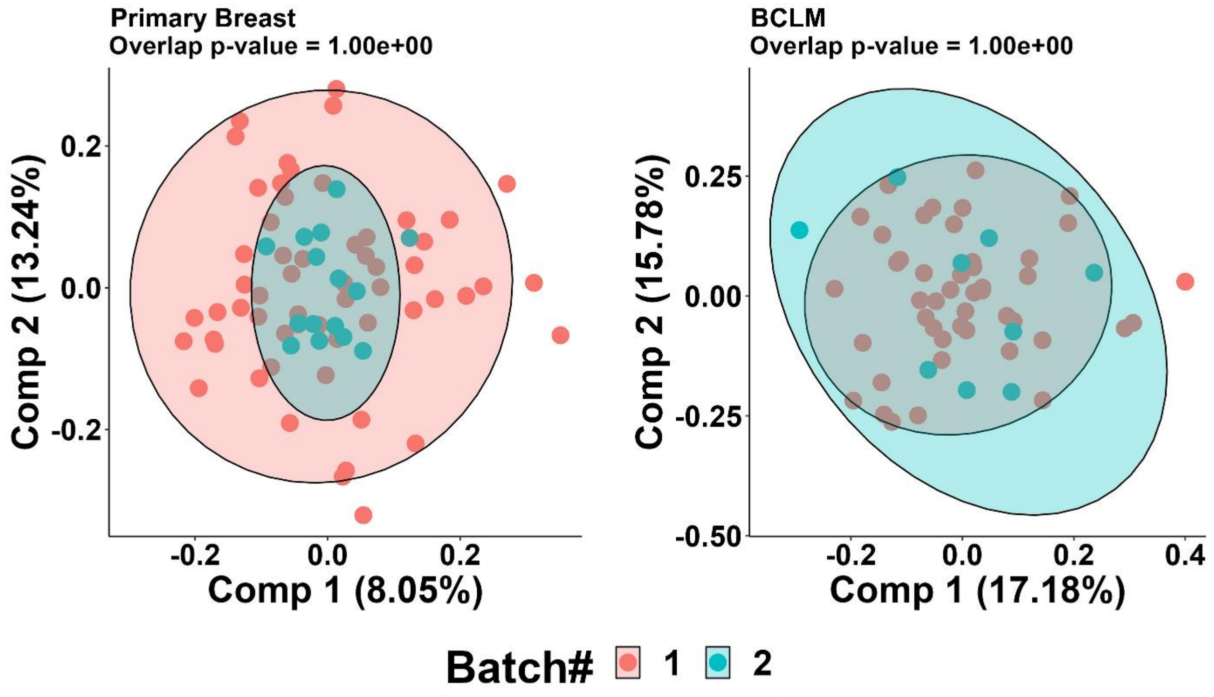

Supplementary Figure 3 – PLS-DA of primary breast and BCLM IMC ROI data by batch number showing that the batches were homogeneous.
