# Supplementary material for: Characterization of the Breast Cancer Liver Metastasis Microenvironment via Machine Learning Analysis of the Primary Tumor Microenvironment
Source: Cancer Res Commun. 2024 Oct 31;4(10):2846–57. doi: 10.1158/2767-9764.CRC-24-0263 (PMC11525956; doi:10.1158/2767-9764.CRC-24-0263)
Supplement: Supplementary Figure S4 — S4. Example of mapping of the annotated phenotype to corresponding segmented cells based on the markers identified by IMC for paired primary tumor and BCLM (patient 15). [file crc-24-0263_supplementary_figure_s4_suppsf4.pdf]

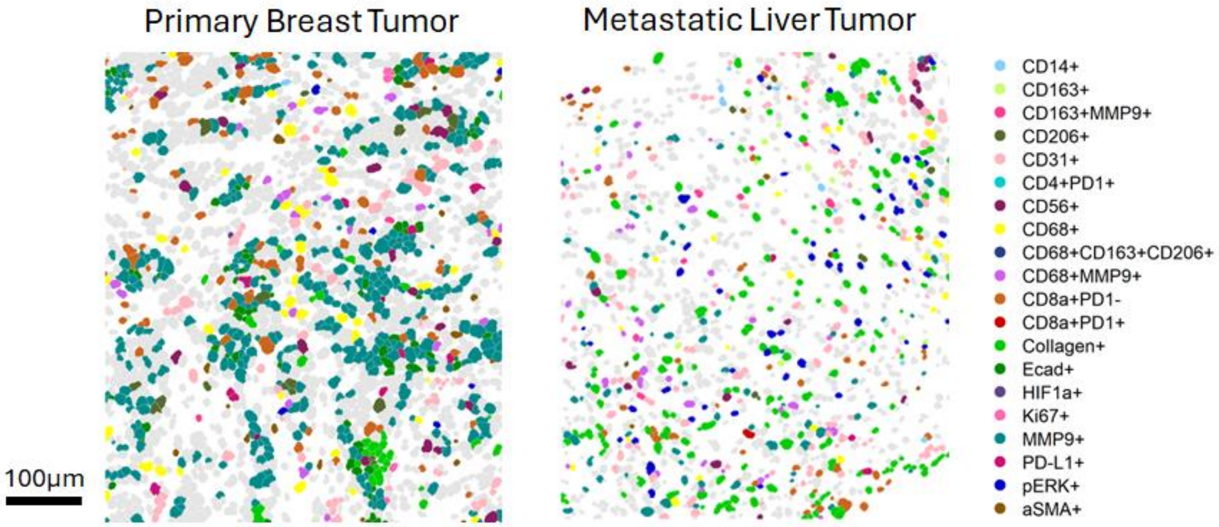

Supplementary Figure 4 – Example of mapping of the annotated phenotype to corresponding segmented cells based on the markers identified by IMC for paired primary tumor and BCLM (patient 15).
