# Supplementary material for: Characterization of the Breast Cancer Liver Metastasis Microenvironment via Machine Learning Analysis of the Primary Tumor Microenvironment
Source: Cancer Res Commun. 2024 Oct 31;4(10):2846–57. doi: 10.1158/2767-9764.CRC-24-0263 (PMC11525956; doi:10.1158/2767-9764.CRC-24-0263)
Supplement: Supplementary Figure S5 — S5. PLS-DA score plots of classifying BCLM IMC cluster densities into Low (<median) or High (≥median) groups using primary tumor IMC cluster densities. [file crc-24-0263_supplementary_figure_s5_suppsf5.pdf]

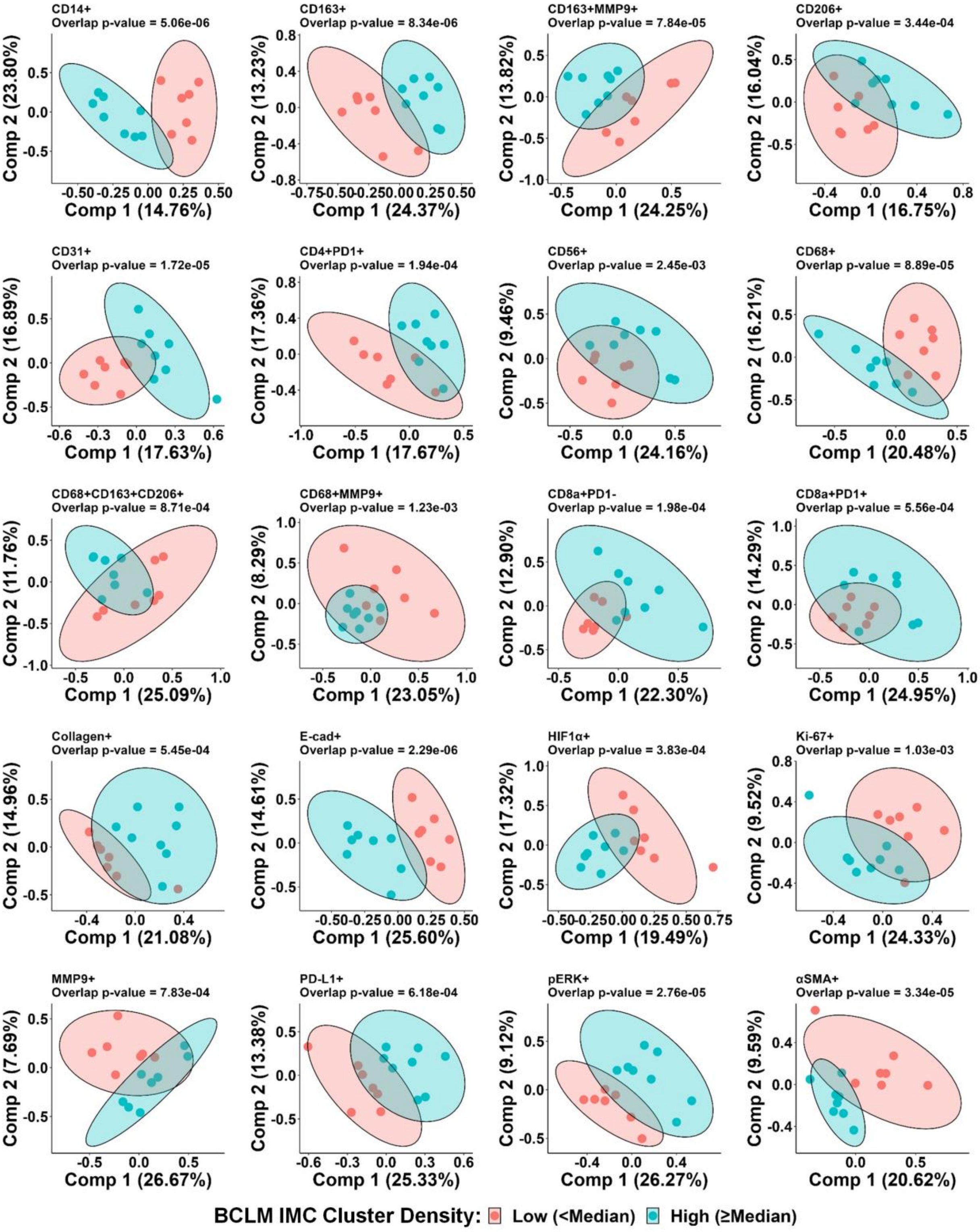

Supplementary Figure 5 – PLS-DA score plots of classifying BCLM IMC cluster densities into Low (<median) or High (≥median) groups using primary tumor IMC cluster densities.
