# Supplementary material for: Characterization of the Breast Cancer Liver Metastasis Microenvironment via Machine Learning Analysis of the Primary Tumor Microenvironment
Source: Cancer Res Commun. 2024 Oct 31;4(10):2846–57. doi: 10.1158/2767-9764.CRC-24-0263 (PMC11525956; doi:10.1158/2767-9764.CRC-24-0263)
Supplement: Supplementary Figure S6 — S6. PLS-DA score plots of classifying BCLM patient IMC clusters into Low (<median) or High (≥median) groups using covariates only. [file crc-24-0263_supplementary_figure_s6_suppsf6.pdf]

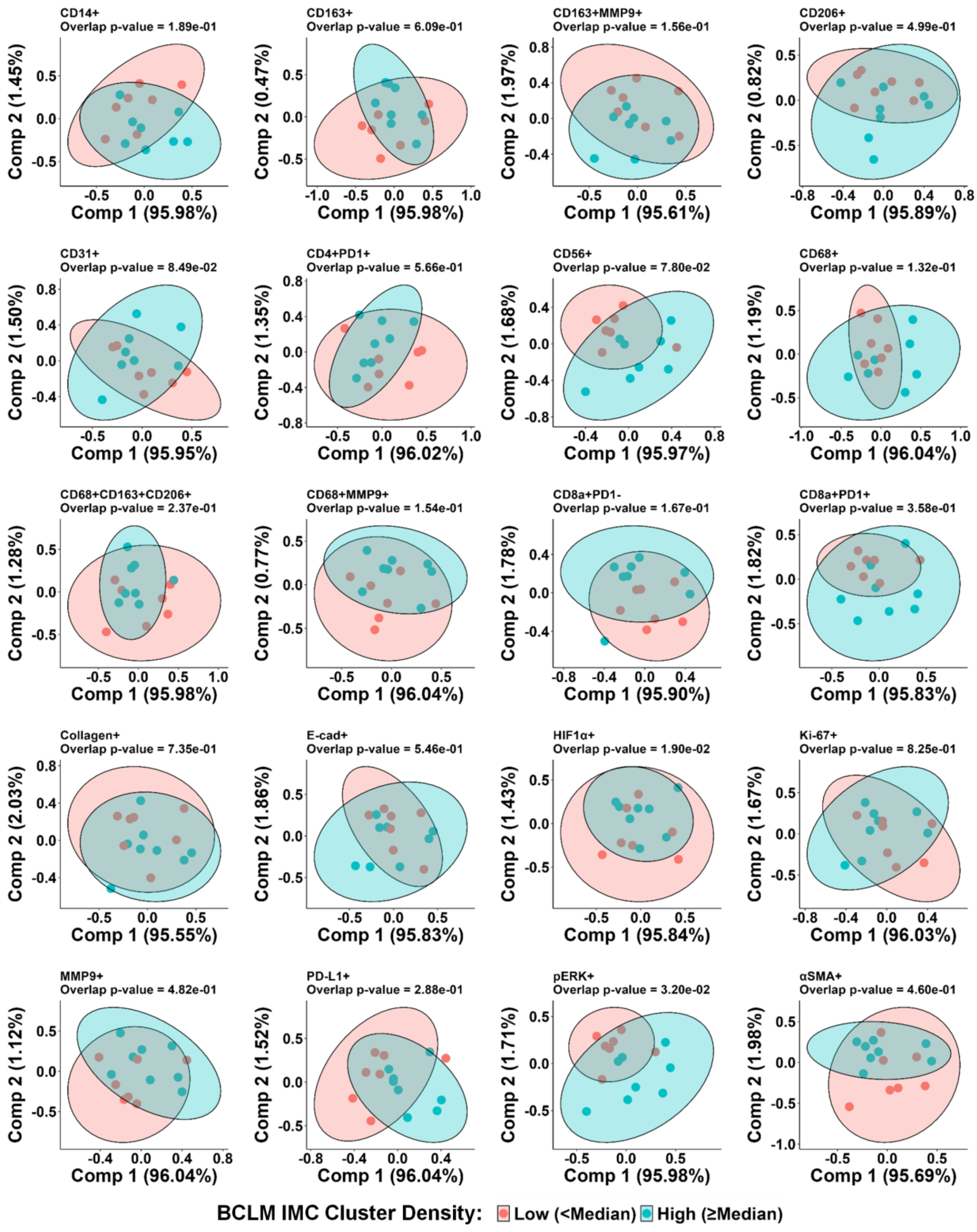

Supplementary Figure 6 – PLS-DA score plots of classifying BCLM patient IMC clusters into Low (<median) or High (≥median) groups using covariates only. Features that were categorical were converted to numerical values (e.g., for ER: negative = 1, weak = 2, medium = 3, and strong = 4).
