# Supplementary material for: Characterization of the Breast Cancer Liver Metastasis Microenvironment via Machine Learning Analysis of the Primary Tumor Microenvironment
Source: Cancer Res Commun. 2024 Oct 31;4(10):2846–57. doi: 10.1158/2767-9764.CRC-24-0263 (PMC11525956; doi:10.1158/2767-9764.CRC-24-0263)
Supplement: Supplementary Figure S8. — S8. Variable importance for prediction of BCLM CD31+, CD4+PD1+, CD56+, and CD68+ using ML models. [file crc-24-0263_supplementary_figure_s8.suppsf8.pdf]

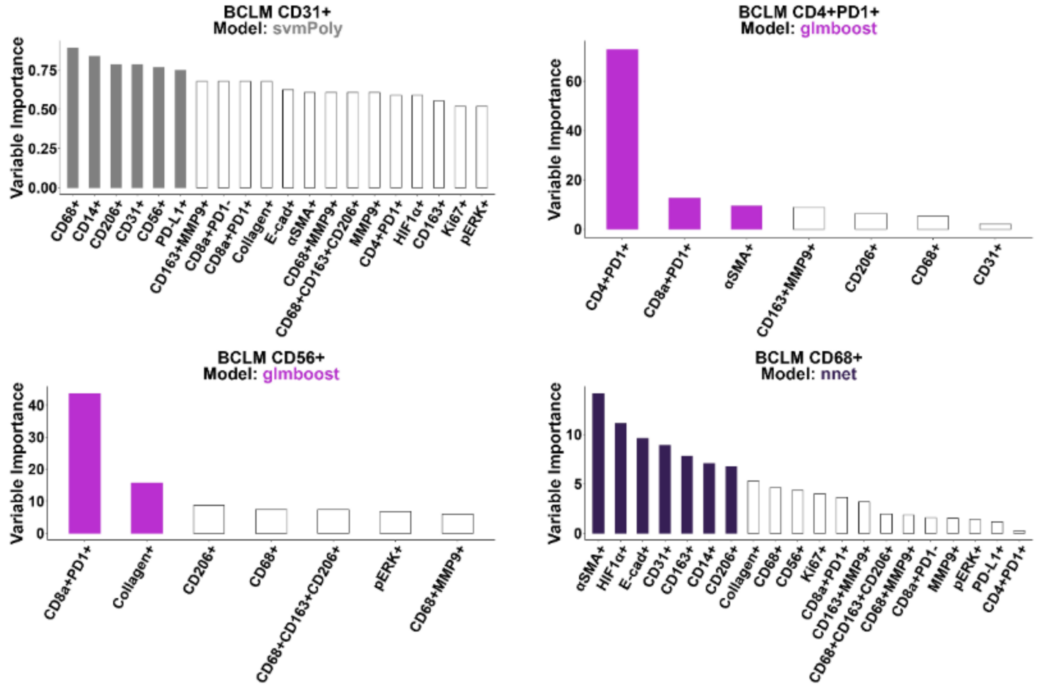

Supplementary Figure 8 – Variable importance for prediction of BCLM CD31+, CD4+PD1+, CD56+, and CD68+ using ML models. IMC clusters given zero variable importance by *varImp* are not shown. Dark bars denote clusters used by the optimized model.
