# Supplementary material for: Characterization of the Breast Cancer Liver Metastasis Microenvironment via Machine Learning Analysis of the Primary Tumor Microenvironment
Source: Cancer Res Commun. 2024 Oct 31;4(10):2846–57. doi: 10.1158/2767-9764.CRC-24-0263 (PMC11525956; doi:10.1158/2767-9764.CRC-24-0263)
Supplement: Supplementary Figure S12 — S12. AUROC achieved by ML models using primary tumor IMC clusters across a variable number of features to predict BCLM IMC cluster densities (as stated in gray box of each panel) [file crc-24-0263_supplementary_figure_s12_suppsf12.pdf]

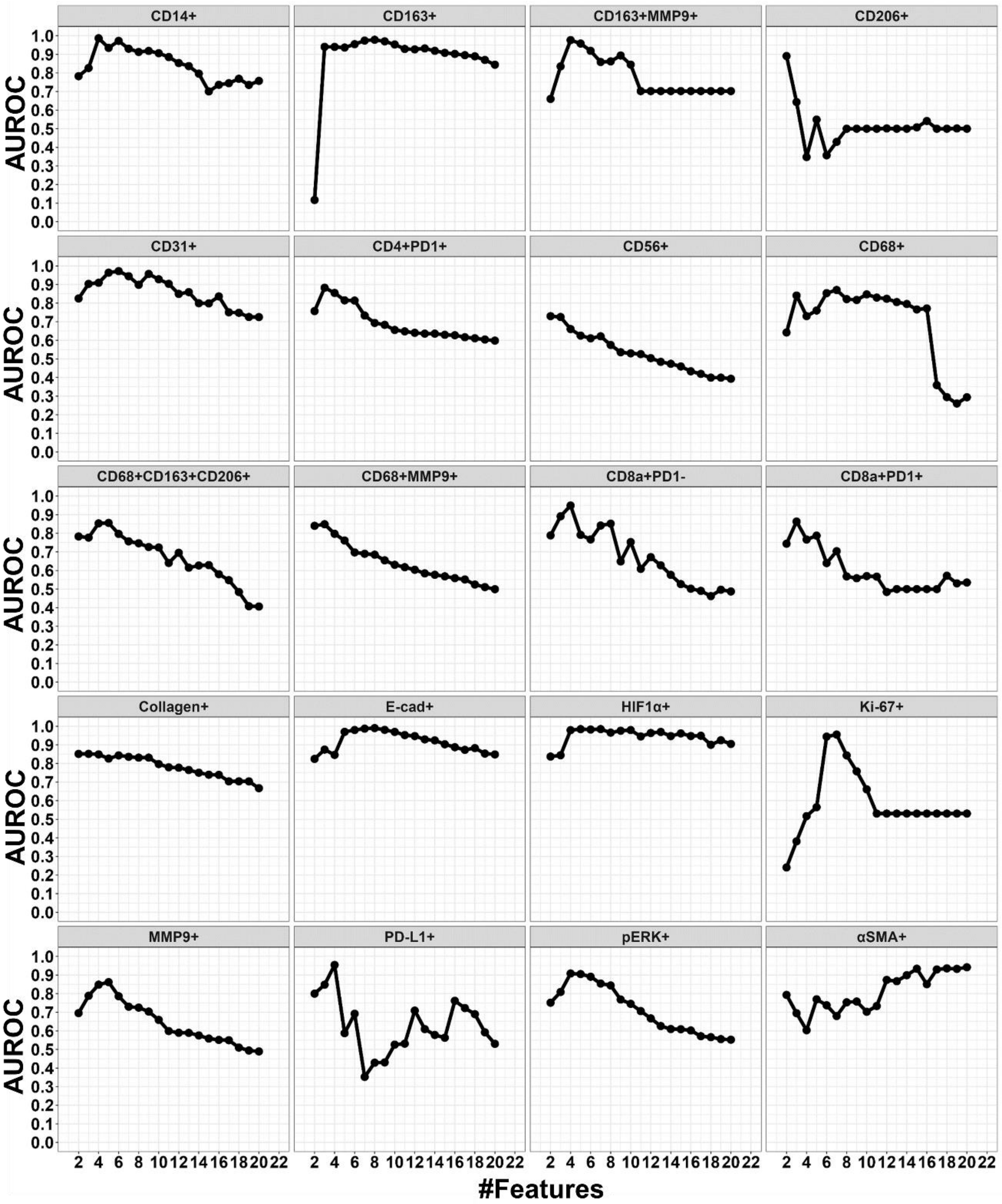

Supplementary Figure 12 – AUROC achieved by ML models using primary tumor IMC clusters across a variable number of features to predict BCLM IMC cluster densities (as stated in gray box of each panel).
