# Supplementary material for: Characterization of the Breast Cancer Liver Metastasis Microenvironment via Machine Learning Analysis of the Primary Tumor Microenvironment
Source: Cancer Res Commun. 2024 Oct 31;4(10):2846–57. doi: 10.1158/2767-9764.CRC-24-0263 (PMC11525956; doi:10.1158/2767-9764.CRC-24-0263)
Supplement: Supplementary Figure S14 — S14. AUROC curves of ML models using covariates only to predict BCLM IMC cluster densities as being either above or below median values. [file crc-24-0263_supplementary_figure_s14_suppsf14.pdf]

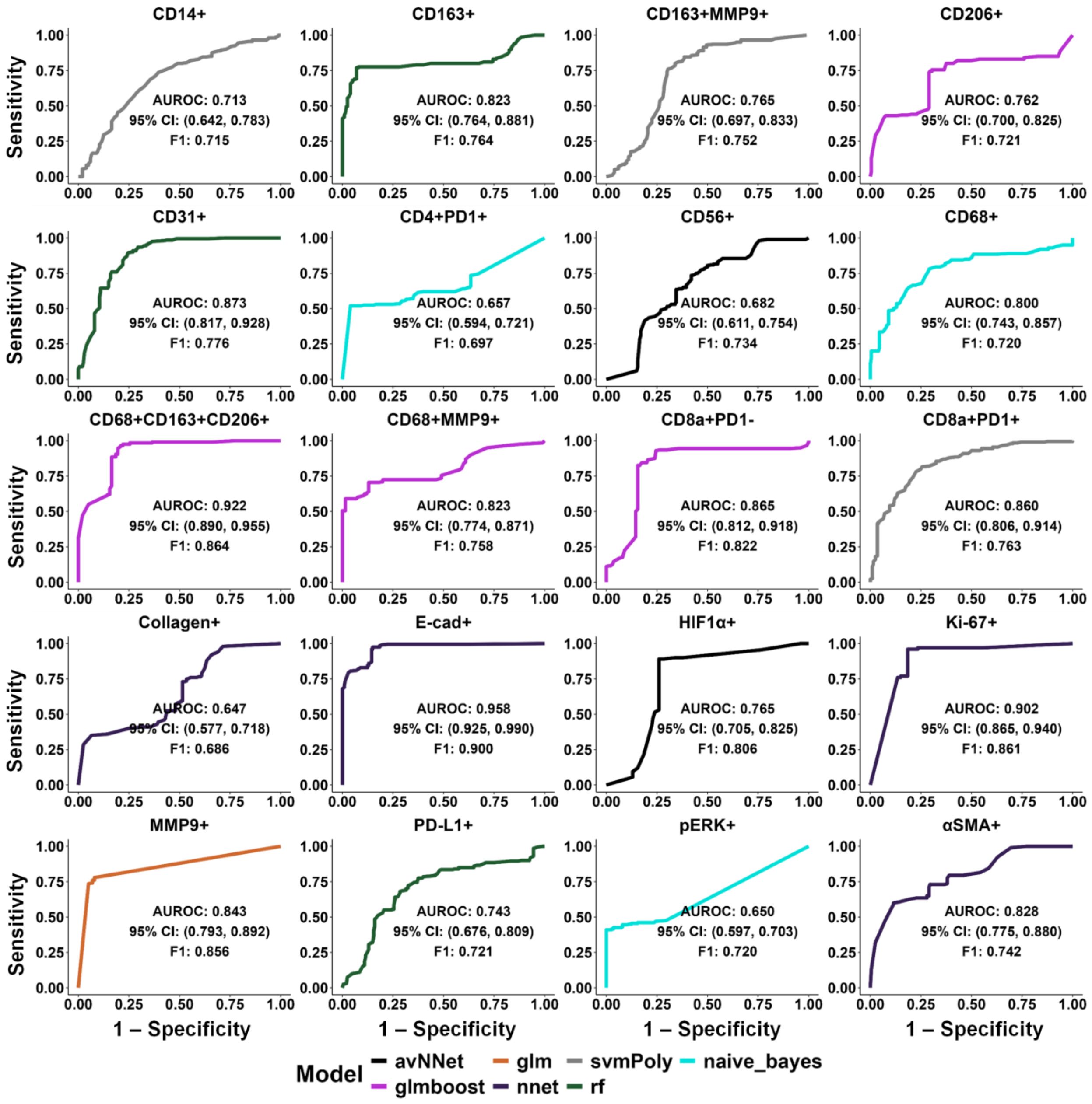

Supplementary Figure 14 – AUROC curves of ML models using covariates only to predict BCLM IMC cluster densities as being either above or below median values. One ML model was created per BCLM cluster.
