# Supplementary material for: Characterization of the Breast Cancer Liver Metastasis Microenvironment via Machine Learning Analysis of the Primary Tumor Microenvironment
Source: Cancer Res Commun. 2024 Oct 31;4(10):2846–57. doi: 10.1158/2767-9764.CRC-24-0263 (PMC11525956; doi:10.1158/2767-9764.CRC-24-0263)
Supplement: Supplementary Table S1 — Table S1. Hyperparameters used for AUROC-optimized Machine Learning models. [file crc-24-0263_supplementary_table_s1_suppst1.pdf]

Supplementary Table 1 – Hyperparameters used for AUROC-optimized Machine Learning models. N/A = Not Applicable.

BCLM Cluster	ML Model Description from *caret*	R Library::R Function	Hyperparameter	Description	Value
CD14+	Least Squares Support Vector Machine with Polynomial Kernel	*kernlab::svmPoly()*	degree	Polynomial Degree	1
			scale	Scale	0.01
			tau	Regularization Parameter	128
CD163+	Neural Network	*nnet::nnet()*	size	#Hidden Units	15
			decay	Weight Decay	0.00750
CD163+MMP9+	Bayesian Generalized Linear Model	*stats::glm()*	N/A	N/A	N/A
CD206+	Model Averaged Neural Network	*nnet:avNNet()*	size	#Hidden Units	5
			decay	Weight Decay	1e-04
			bag	Bagging	FALSE
CD31+	Least Squares Support Vector Machine with Polynomial Kernel	*kernlab::svmPoly()*	degree	Polynomial Degree	3
			scale	Scale	10
			tau	Regularization Parameter	32
CD4+PD1+	Boosted Generalized Linear Model	*mboost::glmboost()*	mstop	# Boosting Iterations	500
			prune	AIC Prune	No
CD56+	Boosted Generalized Linear Model	*mboost::glmboost()*	mstop	# Boosting Iterations	100
			prune	AIC Prune	No
CD68+	Neural Network	*nnet::nnet()*	size	#Hidden Units	3
			decay	Weight Decay	0.000562
CD68+CD163+CD206+	Quantile Random Forest	*randomForest::rf()*	mtry	#Randomly Selected Predictors	2
CD68+MMP9+	Boosted Generalized Linear Model	*mboost::glmboost()*	mstop	# Boosting Iterations	500
			prune	AIC Prune	No
CD8a+PD1+	k-Nearest Neighbors	*caret::knn()*	kmax	Max. #Neighbors	7
			distance	Distance	7
			kernel	Kernel	7

CD8a+PD1-	Neural Networks with Feature Extraction	*nnet::pcaNNet()*	size	#Hidden Units	3
			decay	Weight Decay	0.1
Collagen+	Boosted Generalized Linear Model	*mboost::glmboost()*	mstop	# Boosting Iterations	150
			prune	AIC Prune	No
E-cad+	Oblique Random Forest	*obliqueRF::ORFsvm()*	mtry	#Randomly Selected Predictors	6
HIF1α+	Quantile Random Forest	*randomForest::rf()*	mtry	#Randomly Selected Predictors	3
Ki-67+	Bayesian Generalized Linear Model	*stats::glm()*	N/A	N/A	N/A
MMP9+	Boosted Generalized Linear Model	*mboost::glmboost()*	mstop	# Boosting Iterations	400
			prune	AIC Prune	No
PD-L1+	Model Averaged Neural Network	*nnet:avNNet()*	size	#Hidden Units	1
			decay	Weight Decay	1e-04
			bag	Bagging	False
pERK+	Boosted Generalized Linear Model	*mboost::glmboost()*	mstop	# Boosting Iterations	300
			prune	AIC Prune	no
αSMA+	Least Squares Support Vector Machine with Polynomial Kernel	*kernlab::svmPoly()*	degree	Polynomial Degree	2
			scale	Scale	1000
			tau	Regularization Parameter	64
