# Supplementary material for: Characterization of the Breast Cancer Liver Metastasis Microenvironment via Machine Learning Analysis of the Primary Tumor Microenvironment
Source: Cancer Res Commun. 2024 Oct 31;4(10):2846–57. doi: 10.1158/2767-9764.CRC-24-0263 (PMC11525956; doi:10.1158/2767-9764.CRC-24-0263)
Supplement: Supplementary Table S3 — Table S3. Variable Importance for predicting BCLM CD163+ using primary tumor clusters. [file crc-24-0263_supplementary_table_s3_suppst3.pdf]

Supplementary Table 3 – Variable Importance for predicting BCLM CD163+ using primary tumor clusters. Larger values imply higher variable importance. Clusters used in the optimal model are marked with "X."

Cluster in Primary	Included In Optimal Model	Variable Importance
E-cad+	X	20.253
CD163+	X	12.066
CD68+	X	11.295
CD163+MMP9+	X	9.002
MMP9+	X	7.475
pERK+	X	5.927
CD56+	X	5.530
CD4+PD1+	X	5.306
CD14+		3.569
Ki-67+		3.303
αSMA+		3.185
CD8a+PD1+		3.031
PD-L1+		2.882
CD8a+PD1-		2.301
HIF1α+		1.373
CD31+		1.222
CD68+CD163+CD206+		0.907
CD206+		0.804
CD68+MMP9+		0.539
Collagen+		0.029
