# Supplementary material for: Characterization of the Breast Cancer Liver Metastasis Microenvironment via Machine Learning Analysis of the Primary Tumor Microenvironment
Source: Cancer Res Commun. 2024 Oct 31;4(10):2846–57. doi: 10.1158/2767-9764.CRC-24-0263 (PMC11525956; doi:10.1158/2767-9764.CRC-24-0263)
Supplement: Supplementary Table S4 — Table S4. Variable Importance for predicting BCLM CD163+MMP9+ using primary tumor clusters. [file crc-24-0263_supplementary_table_s4_suppst4.pdf]

Supplementary Table 4 – Variable Importance for predicting BCLM CD163+MMP9+ using primary tumor clusters. Larger values imply higher variable importance. Clusters used in the optimal model are marked with "X."

Cluster in Primary	Included In Optimal Model	Variable Importance
CD206+	X	0.0000511
CD4+PD1+	X	0.0000322
CD68+	X	0.0000281
CD163+	X	0.0000260
MMP9+		0.0000249
CD163+MMP9+		0.0000246
CD8a+PD1+		0.0000240
PD-L1+		0.0000237
CD68+CD163+CD206+		0.0000233
CD68+MMP9+		0.0000229
CD56+		0.0000188
CD14+		0.0000180
CD8a+PD1-		0.0000178
