# Supplementary material for: Characterization of the Breast Cancer Liver Metastasis Microenvironment via Machine Learning Analysis of the Primary Tumor Microenvironment
Source: Cancer Res Commun. 2024 Oct 31;4(10):2846–57. doi: 10.1158/2767-9764.CRC-24-0263 (PMC11525956; doi:10.1158/2767-9764.CRC-24-0263)
Supplement: Supplementary Table S6 — Table S6. Variable Importance for predicting BCLM CD31+ using primary tumor clusters. [file crc-24-0263_supplementary_table_s6_suppst6.pdf]

Supplementary Table 6 – Variable Importance for predicting BCLM CD31+ using primary tumor clusters. Larger values imply higher variable importance. Clusters used in the optimal model are marked with “X.”

Cluster in Primary	Included In Optimal Model	Variable Importance
CD68+	X	0.893
CD14+	X	0.839
CD206+	X	0.786
CD31+	X	0.786
CD56+	X	0.768
PD-L1+	X	0.750
CD163+MMP9+		0.679
CD8a+PD1-		0.679
CD8a+PD1+		0.679
Collagen+		0.679
E-cad+		0.625
αSMA+		0.607
CD68+MMP9+		0.607
CD68+CD163+CD206+		0.607
MMP9+		0.607
CD4+PD1+		0.589
HIF1α+		0.589
CD163+		0.554
Ki-67+		0.518
pERK+		0.518
