# Supplementary material for: Characterization of the Breast Cancer Liver Metastasis Microenvironment via Machine Learning Analysis of the Primary Tumor Microenvironment
Source: Cancer Res Commun. 2024 Oct 31;4(10):2846–57. doi: 10.1158/2767-9764.CRC-24-0263 (PMC11525956; doi:10.1158/2767-9764.CRC-24-0263)
Supplement: Supplementary Table S7 — Table S7. Variable Importance for predicting BCLM CD4+PD1+ using primary tumor clusters. [file crc-24-0263_supplementary_table_s7_suppst7.pdf]

Supplementary Table 7 – Variable Importance for predicting BCLM CD4+PD1+ using primary tumor clusters. Larger values imply higher variable importance. Clusters used in the optimal model are marked with "X."

Cluster in Primary	Included In Optimal Model	Variable Importance
CD4+PD1+	X	72.705
CD8a+PD1+	X	12.524
αSMA+	X	9.352
CD163+MMP9+		8.851
CD206+		6.388
CD68+		5.221
CD31+		2.076
CD14+		0.000
CD68+MMP9+		0.000
CD163+		0.000
CD68+CD163+CD206+		0.000
MMP9+		0.000
CD56+		0.000
CD8a+PD1-		0.000
PD-L1+		0.000
Ki-67+		0.000
pERK+		0.000
HIF1α+		0.000
E-cad+		0.000
Collagen+		0.000
