# Supplementary material for: Characterization of the Breast Cancer Liver Metastasis Microenvironment via Machine Learning Analysis of the Primary Tumor Microenvironment
Source: Cancer Res Commun. 2024 Oct 31;4(10):2846–57. doi: 10.1158/2767-9764.CRC-24-0263 (PMC11525956; doi:10.1158/2767-9764.CRC-24-0263)
Supplement: Supplementary Table S8 — Table S8. Variable Importance for predicting BCLM CD56+ using primary tumor clusters. [file crc-24-0263_supplementary_table_s8_suppst8.pdf]

Supplementary Table 8 – Variable Importance for predicting BCLM CD56+ using primary tumor clusters. Larger values imply higher variable importance. Clusters used in the optimal model are marked with "X."

Cluster in Primary	Included In Optimal Model	Variable Importance
CD8a+PD1+	X	43.623
Collagen+	X	15.671
CD206+		8.660
CD68+		7.337
CD68+CD163+CD206+		7.333
pERK+		6.803
CD68+MMP9+		6.004
CD14+		0.000
CD163+		0.000
CD163+MMP9+		0.000
MMP9+		0.000
CD56+		0.000
CD4+PD1+		0.000
CD8a+PD1-		0.000
PD-L1+		0.000
Ki-67+		0.000
HIF1α+		0.000
E-cad+		0.000
CD31+		0.000
αSMA+		0.000
