# Supplementary material for: Characterization of the Breast Cancer Liver Metastasis Microenvironment via Machine Learning Analysis of the Primary Tumor Microenvironment
Source: Cancer Res Commun. 2024 Oct 31;4(10):2846–57. doi: 10.1158/2767-9764.CRC-24-0263 (PMC11525956; doi:10.1158/2767-9764.CRC-24-0263)
Supplement: Supplementary Table S9 — Table S9. Variable Importance for predicting BCLM CD68+ using primary tumor clusters. [file crc-24-0263_supplementary_table_s9_suppst9.pdf]

Supplementary Table 9 – Variable Importance for predicting BCLM CD68+ using primary tumor clusters. Larger values imply higher variable importance. Clusters used in the optimal model are marked with "X."

Cluster in Primary	Included In Optimal Model	Variable Importance
αSMA+	X	14.142
HIF1α+	X	11.103
E-cad+	X	9.592
CD31+	X	8.898
CD163+	X	7.808
CD14+	X	7.049
CD206+	X	6.721
Collagen+		5.287
CD68+		4.583
CD56+		4.334
Ki-67+		4.003
CD8a+PD1+		3.638
CD163+MMP9+		3.169
CD68+CD163+CD206+		1.972
CD68+MMP9+		1.825
CD8a+PD1-		1.552
MMP9+		1.505
pERK+		1.390
PD-L1+		1.163
CD4+PD1+		0.265
