# Supplementary material for: Characterization of the Breast Cancer Liver Metastasis Microenvironment via Machine Learning Analysis of the Primary Tumor Microenvironment
Source: Cancer Res Commun. 2024 Oct 31;4(10):2846–57. doi: 10.1158/2767-9764.CRC-24-0263 (PMC11525956; doi:10.1158/2767-9764.CRC-24-0263)
Supplement: Supplementary Table S10 — Table S10. Variable Importance for predicting BCLM CD68+CD163+CD206+ using primary tumor clusters. [file crc-24-0263_supplementary_table_s10_suppst10.pdf]

Supplementary Table 10 – Variable Importance for predicting BCLM CD68+CD163+CD206+ using primary tumor clusters. Larger values imply higher importance. Clusters used in the optimal model are marked with "X".

Cluster in Primary	Included In Optimal Model	Variable Importance
CD68+	X	1.226
CD68+MMP9+	X	1.099
CD56+	X	0.972
pERK+	X	0.651
αSMA+	X	0.449
CD163+MMP9+		0.350
HIF1α+		0.228
CD14+		0.221
CD8a+PD1-		0.220
CD68+CD163+CD206+		0.199
CD8a+PD1+		0.198
Ki-67+		0.189
MMP9+		0.165
CD206+		0.157
CD4+PD1+		0.130
Collagen+		0.122
E-cad+		0.110
PD-L1+		0.102
CD31+		0.097
CD163+		0.089
