# Supplementary material for: Characterization of the Breast Cancer Liver Metastasis Microenvironment via Machine Learning Analysis of the Primary Tumor Microenvironment
Source: Cancer Res Commun. 2024 Oct 31;4(10):2846–57. doi: 10.1158/2767-9764.CRC-24-0263 (PMC11525956; doi:10.1158/2767-9764.CRC-24-0263)
Supplement: Supplementary Table S13 — Table S13. Variable Importance for predicting BCLM CD8a+PD1+ using primary tumor clusters. [file crc-24-0263_supplementary_table_s13_suppst13.pdf]

Supplementary Table 13 – Variable Importance for predicting BCLM CD8a+PD1+ using primary tumor clusters. Larger values imply higher variable importance. Clusters used in AUROC-optimized model are marked with "X".

Cluster in Primary	Included In Optimal Model	Variable Importance
CD68+CD163+CD206+	X	0.786
CD8a+PD1+	X	0.768
CD14+	X	0.750
CD163+MMP9+		0.696
CD8a+PD1-		0.696
pERK+		0.679
CD163+		0.661
E-cad+		0.625
CD206+		0.607
CD68+MMP9+		0.589
CD68+		0.571
CD56+		0.571
Ki-67+		0.571
CD4+PD1+		0.554
PD-L1+		0.554
αSMA+		0.554
MMP9+		0.536
HIF1α+		0.536
CD31+		0.536
Collagen+		0.518
