# Supplementary material for: Characterization of the Breast Cancer Liver Metastasis Microenvironment via Machine Learning Analysis of the Primary Tumor Microenvironment
Source: Cancer Res Commun. 2024 Oct 31;4(10):2846–57. doi: 10.1158/2767-9764.CRC-24-0263 (PMC11525956; doi:10.1158/2767-9764.CRC-24-0263)
Supplement: Supplementary Table S15 — Table S15. Variable Importance for predicting BCLM E-cad+ using primary tumor clusters. [file crc-24-0263_supplementary_table_s15_suppst15.pdf]

Supplementary Table 15 – Variable Importance for predicting BCLM E-cad+ using primary tumor clusters. Larger values imply higher variable importance. Clusters used in the optimal model are marked with "X."

Cluster in Primary	Included In Optimal Model	Variable Importance
αSMA+	X	0.911
E-cad+	X	0.857
CD68+MMP9+	X	0.839
HIF1α+	X	0.839
CD68+CD163+CD206+	X	0.830
CD163+MMP9+	X	0.821
CD163+	X	0.732
MMP9+		0.732
CD68+		0.714
CD56+		0.679
Ki-67+		0.679
CD8a+PD1-		0.661
PD-L1+		0.661
CD31+		0.661
CD4+PD1+		0.625
CD206+		0.607
Collagen+		0.589
pERK+		0.571
CD14+		0.554
CD8a+PD1+		0.518
