# Supplementary material for: Characterization of the Breast Cancer Liver Metastasis Microenvironment via Machine Learning Analysis of the Primary Tumor Microenvironment
Source: Cancer Res Commun. 2024 Oct 31;4(10):2846–57. doi: 10.1158/2767-9764.CRC-24-0263 (PMC11525956; doi:10.1158/2767-9764.CRC-24-0263)
Supplement: Supplementary Table S16 — Variable Importance for predicting BCLM HIF1α+ using primary tumor clusters. [file crc-24-0263_supplementary_table_s16_suppst16.pdf]

Supplementary Table 16 – Variable Importance for predicting BCLM HIF1α+ using primary tumor clusters. Larger values imply higher variable importance. Clusters used in the optimal model are marked with "X."

Cluster in Primary	Included In Optimal Model	Variable Importance
CD31+	X	0.790
PD-L1+	X	0.683
CD163+MMP9+	X	0.606
Collagen+	X	0.509
Ki-67+	X	0.421
CD68+	X	0.416
CD8a+PD1-	X	0.411
CD206+		0.340
CD163+		0.309
pERK+		0.274
CD56+		0.270
E-cad+		0.263
CD68+CD163+CD206+		0.254
HIF1α+		0.245
MMP9+		0.208
αSMA+		0.196
CD8a+PD1+		0.187
CD68+MMP9+		0.183
CD4+PD1+		0.178
CD14+		0.167
