# Supplementary material for: Characterization of the Breast Cancer Liver Metastasis Microenvironment via Machine Learning Analysis of the Primary Tumor Microenvironment
Source: Cancer Res Commun. 2024 Oct 31;4(10):2846–57. doi: 10.1158/2767-9764.CRC-24-0263 (PMC11525956; doi:10.1158/2767-9764.CRC-24-0263)
Supplement: Supplementary Table S17 — Table S17. Variable Importance for predicting BCLM Ki-67+ using primary tumor clusters. [file crc-24-0263_supplementary_table_s17_suppst17.pdf]

Supplementary Table 17 – Variable Importance for predicting BCLM Ki-67+ using primary tumor clusters. Larger values imply higher variable importance. Clusters used in the optimal model are marked with "X."

Cluster in Primary	Included In Optimal Model	Variable Importance
CD206+	X	0.0001178
CD4+PD1+	X	0.0001025
MMP9+	X	0.0000995
CD163+	X	0.0000988
PD-L1+	X	0.0000978
CD163+MMP9+	X	0.0000978
CD68+	X	0.0000969
CD14+		0.0000952
CD68+MMP9+		0.0000949
CD8a+PD1+		0.0000933
CD56+		0.0000930
CD68+CD163+CD206+		0.0000904
CD8a+PD1-		0.0000878
