# Supplementary material for: Characterization of the Breast Cancer Liver Metastasis Microenvironment via Machine Learning Analysis of the Primary Tumor Microenvironment
Source: Cancer Res Commun. 2024 Oct 31;4(10):2846–57. doi: 10.1158/2767-9764.CRC-24-0263 (PMC11525956; doi:10.1158/2767-9764.CRC-24-0263)
Supplement: Supplementary Table S18 — Table S18. Variable Importance for predicting BCLM MMP9+ using primary tumor clusters. [file crc-24-0263_supplementary_table_s18_suppst18.pdf]

Supplementary Table 18 – Variable Importance for predicting BCLM MMP9+ using primary tumor clusters. Larger values imply higher variable importance. Clusters used in the optimal model are marked with "X."

Cluster in Primary	Included In Optimal Model	Variable Importance
Collagen+	X	44.789
CD8a+PD1+	X	37.826
CD31+	X	22.642
CD68+	X	15.997
CD56+	X	13.865
CD206+		3.397
MMP9+		2.660
HIF1α+		1.757
E-cad+		1.226
CD14+		0.000
CD68+MMP9+		0.000
CD163+		0.000
CD163+MMP9+		0.000
CD68+CD163+CD206+		0.000
CD4+PD1+		0.000
CD8a+PD1-		0.000
PD-L1+		0.000
Ki-67+		0.000
pERK+		0.000
αSMA+		0.000
