# Supplementary material for: Characterization of the Breast Cancer Liver Metastasis Microenvironment via Machine Learning Analysis of the Primary Tumor Microenvironment
Source: Cancer Res Commun. 2024 Oct 31;4(10):2846–57. doi: 10.1158/2767-9764.CRC-24-0263 (PMC11525956; doi:10.1158/2767-9764.CRC-24-0263)
Supplement: Supplementary Table S21 — Variable Importance for predicting BCLM αSMA+ using primary tumor clusters. [file crc-24-0263_supplementary_table_s21_suppst21.pdf]

Supplementary Table 21 – Variable Importance for predicting BCLM αSMA+ using primary tumor clusters. Larger values imply higher variable importance. Clusters used in the optimal model are marked with "X."

Cluster in Primary	Included In Optimal Model	Variable Importance
E-cad+	X	0.804
HIF1α+	X	0.786
CD68+	X	0.768
PD-L1+	X	0.750
CD8a+PD1+	X	0.696
MMP9+	X	0.679
αSMA+	X	0.679
CD31+	X	0.643
CD8a+PD1-	X	0.607
CD14+	X	0.589
CD206+	X	0.589
CD4+PD1+	X	0.589
pERK+	X	0.589
Collagen+	X	0.589
CD68+CD163+CD206+	X	0.571
Ki-67+		0.536
CD68+MMP9+		0.518
CD163+		0.518
CD56+		0.518
CD163+MMP9+		0.518
