# Supplementary material for: Characterization of the Breast Cancer Liver Metastasis Microenvironment via Machine Learning Analysis of the Primary Tumor Microenvironment
Source: Cancer Res Commun. 2024 Oct 31;4(10):2846–57. doi: 10.1158/2767-9764.CRC-24-0263 (PMC11525956; doi:10.1158/2767-9764.CRC-24-0263)
Supplement: Supplementary Figure S1 — S1. Heatmap of IMC cluster densities originating from breast primary and breast cancer liver metastases (BCLM) before mean aggregation of ROIs. [file crc-24-0263_supplementary_figure_s1_suppsf1.pdf]

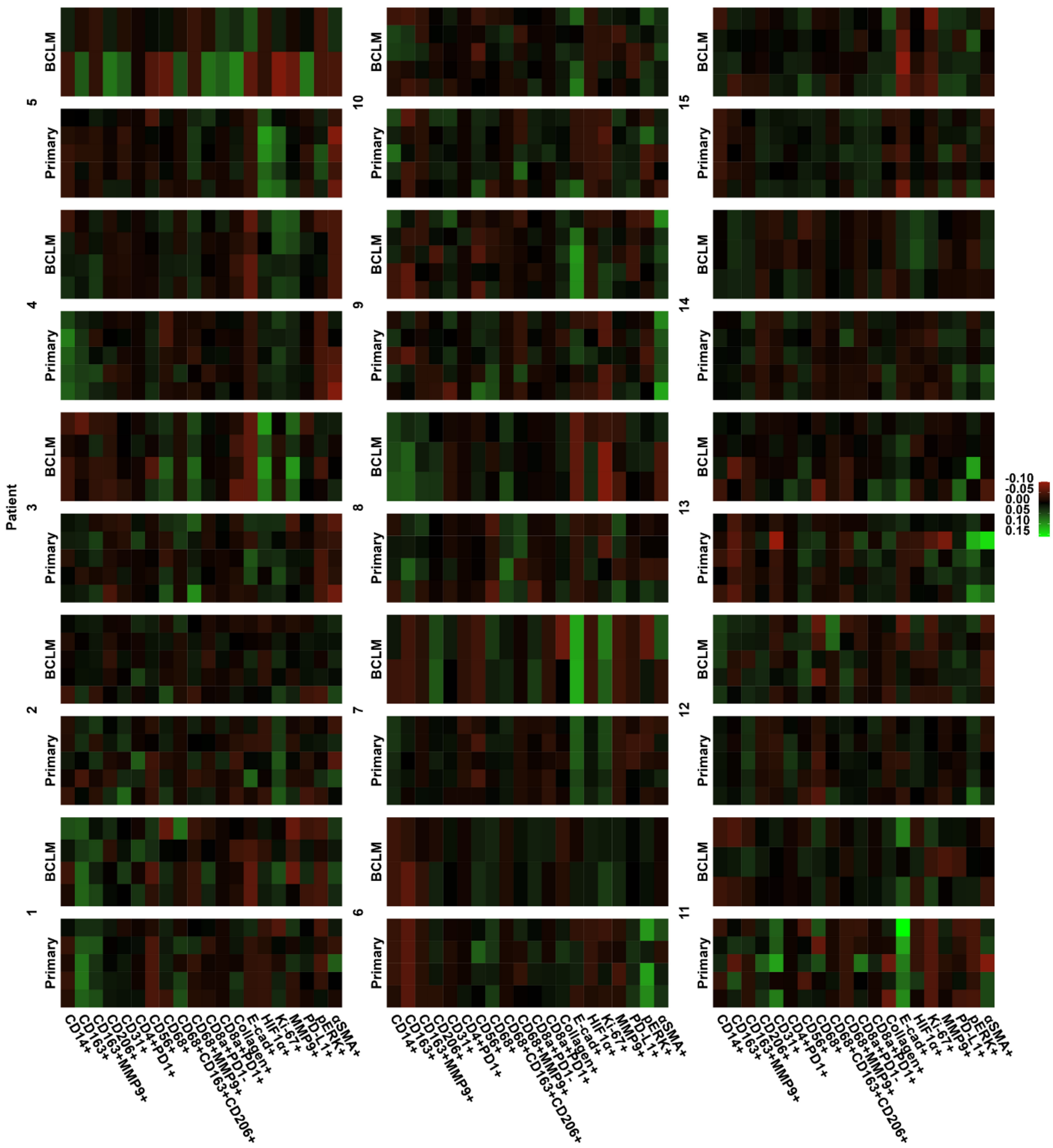

Supplementary Figure 1 – Heatmap of IMC cluster densities originating from breast primary and breast cancer liver metastases (BCLM) before mean aggregation of ROIs.
